# CoCoNest: A continuous structural connectivity-based nested family of parcellations of the human cerebral cortex

**DOI:** 10.1162/netn_a_00409

**Published:** 2024-12-10

**Authors:** Adrian Allen, Zhengwu Zhang, Andrew Nobel

**Affiliations:** Statistics and Operations Research, University of North Carolina at Chapel Hill, Chapel Hill, NC, USA

**Keywords:** Structural connectivity, Diffusion MRI, Multiscale parcellation, Cortical surface, Tree method

## Abstract

Despite the widespread exploration and availability of parcellations for the functional connectome, parcellations designed for the structural connectome are comparatively limited. Current research suggests that there may be no single “correct” parcellation and that the human brain is intrinsically a multiresolution entity. In this work, we propose the Continuous Structural Connectivitity-based, Nested (CoCoNest) family of parcellations—a fully data-driven, multiresolution family of parcellations derived from structural connectome data. The CoCoNest family is created using agglomerative (bottom-up) clustering and error-complexity pruning, which strikes a balance between the complexity of each parcellation and how well it preserves patterns in vertex-level, high-resolution connectivity data. We draw on a comprehensive battery of internal and external evaluation metrics to show that the CoCoNest family is competitive with or outperforms widely used parcellations in the literature. Additionally, we show how the CoCoNest family can serve as an exploratory tool for researchers to investigate the multiresolution organization of the structural connectome.

## Author Summary

In this work, we derive a family of structural connectivity-based parcellations, called the CoCoNest family, based on a continuous representation of structural connectivity. This family is created using agglomerative clustering to grow a fully binary tree and then error-complexity pruning to greedily derive subtrees that balance complexity and goodness of fit. The CoCoNest family is evaluated with a comprehensive collection of internal and external evaluation metrics across two independent datasets and compared with widely used parcellations. Our results show that members of the CoCoNest family are competitive with other parcellations and often achieve superior performance. We show that the CoCoNest family can serve as an exploratory tool for investigations into the multiscale nature of the structural connectome.

## INTRODUCTION

The human connectome has long inspired critical research within the field of neuroscience (Brodmann, 1909; Elam et al., 2021; Hagmann et al., 2008; Leergaard & Bjaalie, 2022; Marin-Padilla, 1983; Sporns, Tononi, & Kötter, 2005; Yeo et al., 2011). The exploration of the human connectome has deepened in recent years due to advances in noninvasive brain imaging techniques. These advances have spurred the collection of massive brain imaging datasets (e.g., the Human Connectome Project (HCP) and the Adolescent Brain Cognitive Development (ABCD) study; Casey et al., 2018; Van Essen et al., 2013), which have enabled and enriched connectome research. A crucial step in connectome research involves parcellating the brain into discrete regions of interest (ROIs) that are both spatially and neurobiologically coherent. This collection of ROIs, also called a parcellation or an atlas, is essential because it reduces the complexity of the connectome data while preserving important features relevant to analyses. Parcellations enable researchers to easily explore the organization of the brain, unravel interactions between brain regions, and study how the connectome relates to the overall brain function and behavior (Glasser et al., 2016; Gosnell, Fowler, & Salas, 2019; Hirsiger et al., 2016; Yeo et al., 2011). Additionally, by viewing the ROIs as nodes and the connection strengths between them as edges, parcellations form a link between human connectome research and the expansive literature on network analysis (Fornito, Zalesky, & Bullmore, 2016; Sporns et al., 2005; J. Wang et al., 2010, 2015).

Connectomes are primarily categorized into two modalities: the functional connectome and the structural connectome. The functional connectome serves as a map of the dynamic organization of the brain, where dependencies between activation patterns, measured using functional MRI (fMRI), are used to quantify the connection between ROIs. The structural connectome serves as an anatomical map, where the presence of interconnecting pathways of white matter fiber tracts, uncovered using diffusion MRI (dMRI) and tractography, are used to quantify the connection between ROIs.

When analyzing the connectome, researchers typically choose between an anatomical and a connectivity-based parcellation. Anatomical-based parcellations (Desikan et al., 2006; Destrieux, Fischl, Dale, & Halgren, 2010) are facilitated by neuroanatomy experts who manually identify anatomical landmarks. These parcellations contain neurobiologically meaningful parcels that are relevant for clinical applications. However, such parcellations are often constructed from small cohorts of subjects that may limit their generalizability. Furthermore, because these parcellations do not directly use connectome data, they may be limited in capturing characteristics unique to the functional or structural connectome. These limitations, along with the collection of massive brain imaging datasets, have motivated the creation of connectivity-based parcellations that directly use functional or structural connectivity data. Recent work in connectivity-based parcellations has predominantly focused on the use of functional connectome data. However, there is a general consensus that the structural architecture of the brain plays a critical role in its functional dynamics (Van Essen, 2013; Passingham, Stephan, & Kötter, 2002). This notion, together with recent advances in structural connectome reconstruction (St-Onge, Daducci, Girard, & Descoteaux, 2018; Van Essen et al., 2013), has inspired deeper explorations of the structural connectome, necessitating the construction of parcellations from structural connectome data.

In this paper, we propose a method for constructing a rich, multiresolution family of parcellations from structural connectome data. This approach leverages recent advances in structural connectome reconstruction to uncover the white matter architecture, explicitly strikes a balance between the complexity of the connectome data and the preservation of high resolution connectivity patterns and facilitates tractable investigations of the multiresolution nature of the structural connectome. To achieve this, we start by leveraging a recently developed tractography algorithm called surface-enhanced tractography (SET), which has been shown to decrease gyral bias and better approximate the underlying white matter structure (St-Onge et al., 2018). Subsequently, we use a continuous representation of structural connectivity (SC) (Cole et al., 2021; Consagra, Cole, & Zhang, 2022; Gutman et al., 2014; Mansour L., Seguin, Smith, & Zalesky, 2022; Moyer, Gutman, Faskowitz, Jahanshad, & Thompson, 2017), which provides a rigorous statistical framework for modeling white matter fiber track endpoints and constructs dense, high-resolution SC matrices. Starting with the high-resolution SC data, we use a conventional agglomerative (bottom-up) clustering algorithm to construct a full binary tree that aims to reflect the hierarchical organization of the structural connectome. We then use error-complexity pruning (Breiman, Friedman, Olshen, & Stone, 1984; Chou, Lookabaugh, & Gray, 1989) to iteratively remove branches from this tree in a greedy fashion, balancing the complexity of the tree with its fit to the high-resolution connectome data. This procedure creates a nested sequence of subtrees where each subtree corresponds to a member of our multiresolution parcellation family. This allows users not only to choose the desired complexity of the parcellation but also to explore the interactions and distinctions between multiple resolutions of the structural connectome. In addition, we draw on a collection of internal and external evaluation metrics from the literature to assess the consistency of our parcellation with neurobiological intuition and its performance in typical downstream tasks across two independent datasets.

Our internal and external evaluation results demonstrate that members of our proposed family of parcellations, which we call the CoCoNest family, are competitive or outperform several widely used parcellations in the literature. Additionally, we make CoCoNest easy to reconstruct and access in the most popular template spaces. The code and data used to create the CoCoNest family are freely available at https://github.com/sbci-brain/CoCoNest.

## MATERIALS AND METHODS

**Figure 1.**
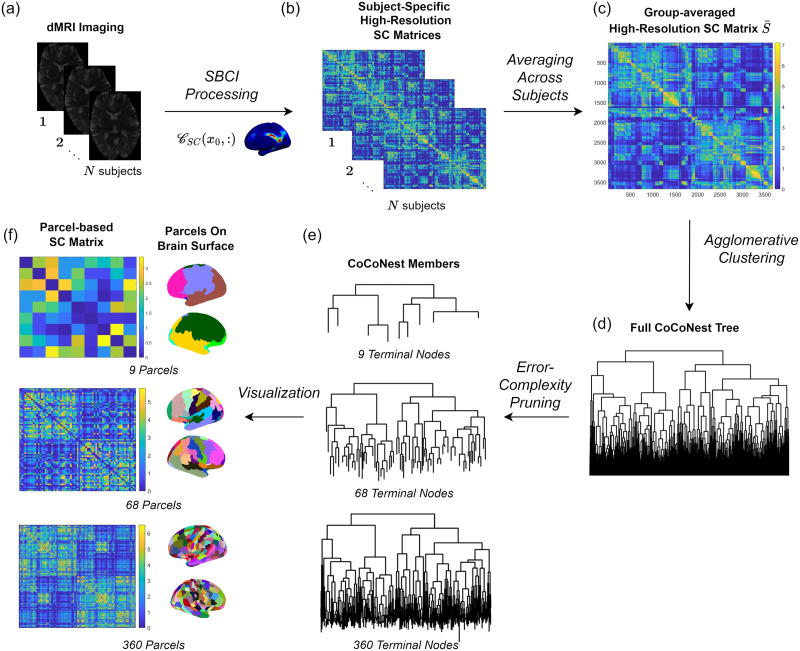
Pipeline for creating the CoCoNest family of parcellations. (A) The first step in the pipeline is acquiring high-quality dMRI imaging data from a homogeneous group of subjects (such as the HCP). (B) The dMRI data are then fed into a recently developed neuroimaging pipeline called the SBCI pipeline, which outputs subject-specific, high-resolution SC matrices by leveraging a continuous model of SC. (C) These connectivity matrices are then averaged together to create a group-averaged, high-resolution SC matrix, ${\bar{S}}^{\prime }$. (D) Next, ${\bar{S}}^{\prime }$ is fed into an agglomerative clustering algorithm to produce a full binary tree. (E) Subsequently, an error-complexity pruning algorithm is used to create a nested, multiresolution sequence of parcellations. (F) Each member of the CoCoNest family can be displayed on the brain’s surface and used in SC analyses.

Figure 1 illustrates the pipeline used to create the CoCoNest family. We began by downloading high-quality imaging data from the HCP (Van Essen et al., 2013). These imaging data were processed using a comprehensive pipeline called the Surface-Based Connectivity Integration (SBCI) pipeline, recently developed by Cole et al. (2021). For each subject, SBCI performed standard image preprocessing steps and outputted a high-resolution SC matrix based on a continuous representation of SC. Each element of these matrices quantifies the density of white matter fiber tracts between two vertices on the brain’s surface. These subject-specific, high-resolution SC matrices were then averaged to obtain a high-resolution, group-averaged SC matrix, denoted as ${\bar{S}}^{\prime }$. ${\bar{S}}^{\prime }$ was then fed into an agglomerative clustering algorithm, which created a full binary tree. This tree aims to both group together vertices with similar SC patterns and capture the multiresolution nature of the SC data in ${\bar{S}}^{\prime }$. Finally, we pruned this tree by adopting an error-complexity pruning approach, developed by Breiman et al. (1984). This pruning procedure resulted in the nested, multiresolution sequence of parcellations that we call the CoCoNest family. The sections below explore each step in more detail.

### Data and Preprocessing

#### Data.

CoCoNest was constructed using high-quality dMRI and structural MRI (sMRI) data from 897 healthy, young adults (ages 22–35 years) who participated in the HCP (Van Essen et al., 2013). The HCP provides rich subject-level data on brain connectivity, behavior, and genetics. Minimally preprocessed data from this study can be accessed at https://db.humanconnectome.org/. Details on the data collection and the minimal preprocessing steps can be found in Van Essen et al. (2013).

#### Image data processing and connectome extraction.

The dMRI and sMRI data were processed through a recently developed neuroimaging pipeline called the SBCI pipeline (Cole et al., 2021). This pipeline consists of dMRI preprocessing, cortical surface extraction, and structural connectome reconstruction steps. A brief overview of these steps is provided below.

Starting with minimally preprocessed dMRI and sMRI data, SBCI follows standard image preprocessing steps. The dMRI data are skull stripped, bias-field corrected, cropped, intensity normalized, and resampled to 1-mm isotropic resolution using tools from MRtrix3, Advanced Normalization Tools (ANTs), and the Sherbrooke Connectivity Imaging Lab toolbox (Scilpy). Subsequently, the sMRI data are registered to the dMRI space and then processed using FreeSurfer’s recon-all. SBCI then computes voxel-wise fiber orientation distribution function (fODF) estimates, using Dipy’s implementation of constrained spherical deconvolution (Tournier, Calamante, & Connelly, 2007). These fODF estimates are then fed into a recently developed probabilistic tractography algorithm called SET (St-Onge et al., 2018), which incorporates information about the geometry of the white surface to improve tractography results. The SET algorithm has been shown to reduce gyral bias in SC and limit the amount of false-positive connections output from tractography.

**Figure 2.**
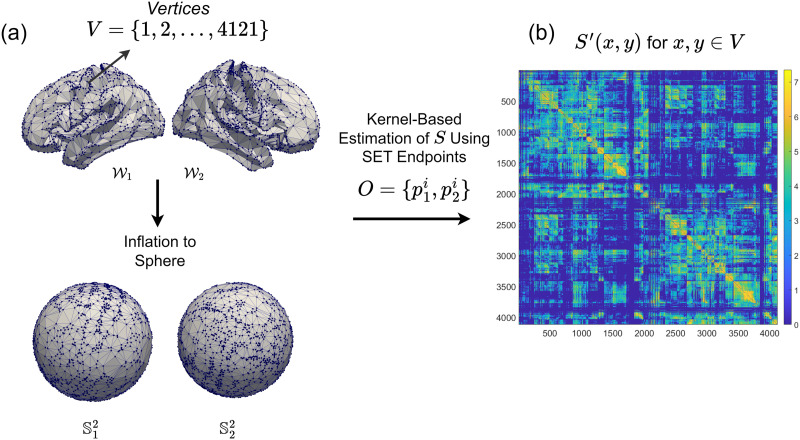
(A) The white matter surface meshes and their inflation to spheres, which are utilized in the CoCoNest pipeline. These meshes contain 4,121 vertices on the brain’s surfaces. A kernel-based algorithm is employed to estimate the continuous structural connectivity function *S* from the endpoints *O* derived from SET. (B) The continuous structural connectivity as a 4,121 × 4,121 matrix after the log transformation.

Let $\Omega =W_{1}\cup W_{2}$ be the union of two white matter surfaces, $W_{1}$ and $W_{2}$, corresponding to the left and right hemispheres of the brain (as shown in the top panel of Figure 2A). In this context, the set Ω × Ω represents all possible pairs of endpoints for white matter tracts that interface with the white matter boundary. The output of the SET algorithm is then a subset of Ω × Ω, denoted by $\tilde{O}=\{\left({\tilde{p}}_{1}^{1},{\tilde{p}}_{2}^{1}),\ldots ,({\tilde{p}}_{1}^{q},{\tilde{p}}_{2}^{q}\right)\}$, where each pair of points $\left({\tilde{p}}_{1}^{i},{\tilde{p}}_{2}^{i}\right)$ indicates the ending positions of white matter tracts on Ω, and *q* is the total number of endpoint pairs. Since $W_{1}$ and $W_{2}$ are homeomorphic to $S^{2}$, SBCI parametrizes them using spherical coordinates. Let (*p*_1_, *p*_2_) be the image of $\left({\tilde{p}}_{1},{\tilde{p}}_{2}\right)$ on $S_{1}^{2}\cup S_{2}^{2}$ under the homeomorphism. Then, we can denote the set of reparameterized ending points as $O=\left\{\left(p_{1}^{1},p_{2}^{1}\right),\ldots ,\left(p_{1}^{q},p_{2}^{q}\right)\right\}$, and with a slight abuse of notation, we let $\Omega =S_{1}^{2}\cup S_{2}^{2}$, as shown in the bottom panel of Figure 2A.

Following the construction of Consagra, Cole, Qiu, and Zhang (2023), for a single subject, SBCI models the streamline endpoints *O* as an outcome of a stochastic point process on Ω × Ω with an integrable but unknown intensity function *S*: Ω × Ω → [0, ∞). This function is defined as follows: For any two measurable regions *E*_1_ ⊂ Ω and *E*_2_ ⊂ Ω, let *N*(*E*_1_, *E*_2_) be the number of streamlines ending in (*E*_1_, *E*_2_). Then, *S* satisfies the following equation:

$$E\left[N\left(E_{1},E_{2}\right)\right]=\int_{E_{1}}\int_{E_{2}}S\left(x,y\right)\;dx\;dy<\infty .$$ (1)

The latent function *S* is referred to as continuous connectivity and is considered in multiple works (Cole et al., 2021; Consagra et al., 2022; Mansour L. et al., 2022; Moyer, Gutman, Faskowitz, et al., 2017). In SBCI, a kernel-based algorithm is employed to estimate *S* from the endpoints *O*. The white surfaces output by FreeSurfer contain over 120,000 vertices. To reduce computational complexity, down-sampled white matter surfaces (see Figure 2A) with *M* = 4, 121 vertices are created by minimizing the induced Hausdorff distance between the full and down-sampled meshes (Cole et al., 2021). SBCI then computes function values *S*(*x*, *y*) for *x* and *y* in the 4,121 downsampled vertices (see Figure 2B for one example of *S*).

In our parcellation pipeline, we excluded from the matrix *S* 446 vertices that correspond to the corpus callosum, as identified by Glasser et al. (2016), as the corpus callosum is often treated separately in connectivity analyses.

Thus, we obtained a 3,675 × 3,675 high-resolution SC matrix for each of the 897 subjects in HCP. The connectivity values in these matrices were highly skewed, with a large number of weak connections and very few strong connections. To prevent our method, and several of the evaluation metrics, from being dominated by these extreme values, we scaled and log-transformed each of the connectivity values, *S*′ = log ((10^5^ × *S* + 1). These transformed SC matrices were then averaged to produce a high-resolution, group-level SC matrix, denoted ${\bar{S}}^{\prime }$ (see Figure 1C). Averaging serves to enhance the signal-to-noise ratio of the SC values and yields a group-level SC representation.

For more details on the SBCI pipeline, see Cole et al. (2021) and the accompanying Github repositories at https://github.com/sbci-brain/.

### Tree Creation

**Figure 3.**
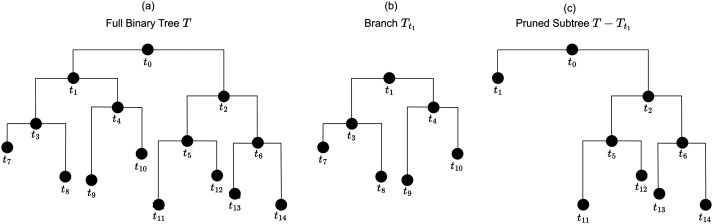
An example of a full binary tree (or dendrogram). (A) A full binary tree *T* rooted at the root node {*t*_0_} along with six internal nodes {*t*_1_, *t*_2_, *t*_3_, *t*_4_, *t*_5_, *t*_6_} and eight terminal nodes {*t*_7_, *t*_8_, *t*_9_, *t*_10_, *t*_11_, *t*_12_, *t*_13_, *t*_14_}. An example of the branch *T*_*t*_1__ rooted at node *t*_1_ is shown in (B). *T*_*t*_1__ consists of the node *t*_1_ along with all of its descendant nodes. (C) An example of *T*_*t*_1__ being pruned from *T*. Pruning a branch *T*_*t*_ from *T* corresponds to collapsing all nodes below *t* and making *t* a terminal node.

Using ${\bar{S}}^{\prime }$, we measured the similarity of SC patterns across the cortical surface, at multiple levels of granularity, that is, resolutions. At the highest resolution, we considered the similarity between SC patterns at the level of individual vertices. We then iteratively merged these vertices into parcels to measure the similarity of SC patterns at coarser resolutions. This was carried out using a standard agglomerative clustering algorithm. In the first stage of this algorithm, each of the 3,675 vertices is assigned to its own distinct parcel. In subsequent stages, the most similar parcels are merged, until all parcels are merged into a single parcel. The result of this algorithm is a full binary tree, or dendrogram, where each parcel has either zero or two children (Rosen, 2011). The final parcel, with no parent, is called the root node; parcels with two children are called internal nodes, while parcels with no children are called terminal nodes. Figure 3A illustrates these concepts.

We quantified the similarity between two parcels *E*_*i*_ and *E*_*j*_ using the average linkage function, defined as follows:

$$D\left(E_{i},E_{j}\right)=\frac{1}{|E_{i}\|E_{j}|}\sum_{x\in E_{i}}\sum_{y\in E_{j}}d\left(x,y\right),$$ (2)

where ∣*E*_*i*_∣ and ∣*E*_*j*_∣ represent the number of vertices in parcels *E*_*i*_ and *E*_*j*_, respectively, and *d*(*x*, *y*) is the similarity between vertices *i* and *j*. The similarity *d*(*x*, *y*) was measured using the Euclidean distance between the SC profiles of vertex *x* and vertex *y*,

$$d\left(x,y\right)=\sqrt{\sum_{z=1}^{3,675}{\left({\bar{S}}^{\prime }\left(x,z\right)-{\bar{S}}^{\prime }\left(y,z\right)\right)}^{2}}.$$ (3)

This similarity captures the intuition that the two vertices are similar if they have similar SC to other regions, and dissimilar if their connectivity to other regions are substantially different. Thus, at each stage of the algorithm, the two parcels with the highest similarity, or the smallest *D*(*E*_*i*_, *E*_*j*_), were merged. We found that the average linkage function and Euclidean distance metric performed better than other popular choices, for example, Ward’s linkage, centroid linkage, and correlation distance (see Supporting Information Section S2). Figure 1D shows a visualization of the full binary tree constructed using the average linkage function and Euclidean distance metric. Throughout the rest of the paper, we call this tree the full CoCoNest tree and denote it as *T*_max_.

### Tree Pruning

In order to derive a family of parcellations from *T*_max_, we make use of tree pruning—a popular technique that has been widely studied in classification and regression trees, vector quantization, and signal compression (Breiman et al., 1984; Chou et al., 1989; Gersho & Gray, 1991; Quinlan, 1993).

We define the branch *T*_*t*_ as the subset of *T*_max_ consisting of the node *t* and all of its descendant nodes. To prune *T*_*t*_ from *T*_max_, we delete all descendants of *t* from *T*_max_, leaving *t* itself in place. After pruning, *t* becomes a terminal node, and a new tree *T*_max_ − *T*_*t*_ is formed. Figure 3C illustrates a single branch being pruned from a tree. If a tree *T* is obtained by iteratively pruning branches from *T*_max_, then *T* is called a “pruned subtree” of *T*_max_, and this relationship is written as *T* ≼ *T*_max_. A parcellation is derived from a pruned subtree *T* by treating the vertices in a terminal node of *T* as a distinct parcel. Thus, by iteratively pruning *T*_max_, we can create a set of parcellations.

A dendrogram visually represents the results of a hierarchical clustering algorithm: The height of each node indicates the similarity between the two clusters, called parcels in this paper, that are being merged at that node. The most popular method of tree pruning is carried out by horizontally “cutting” the dendrogram at a fixed height, that is, collapsing all of the nodes below this fixed height into terminal nodes. This cutoff height effectively sets a limit on the maximum *D*(*E*_*i*_, *E*_*j*_) allowable for merging parcels. The limit is often determined by the desired number of parcels, that is, the user will choose a cutoff height such that no more than *K* parcels remain.

However, this form of tree pruning does not actively balance the core goals of a parcellation: reducing the complexity of the connectome data while preserving meaningful patterns in the data. Instead, to prune *T*_max_, we adopt the error-complexity pruning method introduced by Breiman et al. (1984) and further generalized by Chou et al. (1989). This method iteratively prunes a tree in a greedy fashion, reducing its complexity while seeking to minimize its loss of fit, or error, to the data. We will first discuss how we quantify the fit and complexity of a tree *T* *T*_max_; then, using these concepts, we will provide a brief overview of how we carry out error-complexity pruning to derive a nested, multiresolution family of parcellations.

To measure how well a tree *T* fits the high-resolution SC data in ${\bar{S}}^{\prime }$, we first quantify the fit of a node *t* ∈ *T*. Let *V*_*t*_ be the set of vertices on the brain surface that are merged into node *t* and let *n*_*t*_ be the number of vertices in *V*_*t*_. To evaluate how well the node *t* fits the high-resolution SC data in ${\bar{S}}^{\prime }$, we consider the average SC between the vertices in *V*_*t*_, ${\bar{s}}_{t}=\sum_{x,y\in V_{t}}{\bar{S}}^{\prime }(x,y)/(2\times (\begin{array}{c} n_{t}\\ 2 \end{array}))$ (with the convention that ${\bar{S}}^{\prime }\left(x,x\right)=0$) and the average SC between a vertex in *V*_*t*_ and a vertex *y* not in *V*_*t*_, ${\bar{s}}_{t,y}=\sum_{x\in V_{t}}{\bar{S}}^{\prime }\left(x,y\right)/n_{t}$. With these definitions, we define the fit of *t* as follows:

$$R\left(t\right)=\sum_{x,y\in V_{t}}\|{\bar{S}}^{\prime }(x,y)-{\bar{s}}_{t}{\|}^{2}+\sum_{x\in V_{t}}\sum_{y\notin V_{t}}\|{\bar{S}}^{\prime }(x,y)-{\bar{s}}_{t,y}{\|}^{2}.$$ (4)

The quantity *R*(*t*) measures the error between ${\bar{S}}^{\prime }$ and a lower resolution SC obtained by treating the vertices in *V*_*t*_ as a single parcel and averaging their SC patterns. More specifically, the first term quantifies the error incurred when the average SC within *V*_*t*_ is used as a substitute for the original connections in ${\bar{S}}^{\prime }$. Similarly, the second term quantifies the error incurred when the SC between a vertex in *V*_*t*_ and a vertex outside *V*_*t*_ is set to the average SC of all vertices in *V*_*t*_ to that external vertex. The fit of *T* *T*_max_ to ${\bar{S}}^{\prime }$ is then obtained by adding together the fits of its terminal nodes,

$$R\left(T\right)=\sum_{t\in \tilde{T}}R\left(t\right),$$ (5)

where $\tilde{T}$ denotes the set of terminal nodes in *T*. The fit, or error, of the full tree *R*(*T*_max_) is 0, reflecting the fact that each terminal node of *T*_max_ contains a single vertex in ${\bar{S}}^{\prime }$. We measure the complexity of a tree *T* *T*_max_ by its number of terminal nodes, $|\tilde{T}|$. Thus the low error of *T*_max_ is offset by its high complexity. The error-complexity measure of *T* is given by the following:

$$R_{\alpha }\left(T\right)=R\left(T\right)+\alpha |\tilde{T}|,$$ (6)

where *α* ≥ 0 balances the error and complexity of *T*. For a given *α*, error-complexity pruning seeks to find a subtree *T* ≺ *T*_max_ that minimizes *R_α_*(*T*). If *α* is 0, then the full tree *T*_max_ will minimize *R*_0_(*T*). As the complexity parameter *α* increases, the minimizing subtree *T* will have fewer terminal nodes. For a sufficiently large *α*, the minimizing subtree will simply be the root node of *T*_max_.

Since it is computationally infeasible to explore every combination of pruning decisions to find the subtree that minimizes *R_α_*(*T*), a method called weakest link cutting is used to facilitate error-complexity pruning.

At each stage of the pruning algorithm, weakest link cutting prunes the branch *T*_t*_ that leads to the smallest decrease in the error-complexity function, *R_α_*(*T* − *T*_*t*_) − *R_α_*(*T*). This is equivalent to pruning the branch *T*_t*_ that minimizes the following:

$$\frac{R\left(T\right)-R\left(T-T_{t}\right)}{|T-T_{t}|-|T|},$$ (7)

where only the branches rooted at nonterminal nodes are considered. This minimizing branch is called the weakest link since it has the smallest impact on the change in the error-complexity function.

*T*_t*_ is then pruned to create a new tree *T*_1_ = *T*_max_ − *T*_t*_. Subsequently, the method finds a new weakest link in *T*_1_ and prunes the corresponding branch. This process is repeated until only the root node remains. More details on this algorithm can be found in Breiman et al. (1984).

The result of this algorithm is a nested sequence of subtrees *T*_max_ ≻ *T*_1_ ≻ *T*_2_ … root. The terminal nodes of each each subtree in this sequence yields a distinct parcellation of the cortical surface. We call the resulting nested, multiresolution sequence of parcellations the CoCoNest family. The pruning procedure tends to remove two terminal nodes at a time during the early stages of the algorithm, while larger cuts are made in the later stages, as illustrated in Figure 9. Thus, although a parcellation of any fixed size (number of terminal nodes) *K* is not guaranteed, the CoCoNest sequence still spans a wide range of resolutions.

### Quantifying Parcellation Performance

In this section, we use several metrics for quantifying the quality of a given parcellation. We divide these metrics into two categories: internal evaluation and external evaluation. Internal evaluation metrics measure the performance of a parcellation based on properties intrinsic to the parcels themselves, such as the size of a parcel, without reference to external information. In contrast, external evaluation metrics rely on auxiliary or external information, like a subject’s performance on a cognitive test. In the Results section, we used these metrics to assess the performance of members of the CoCoNest family and to compare its performance with other parcellations commonly used in the literature.

Several of the metrics are based on the idea of converting a high-resolution SC matrix to a parcel-based SC matrix. Let $A=\left\{E_{1},E_{2},\ldots E_{K}\right\}$ denote a parcellation of Ω with *K* parcels. We can define the connectivity strength between parcels *E*_*i*_ and *E*_*j*_ as the average number of streamlines per unit area square. This is approximated through the equation:

$$S_{A}^{\prime }\left(E_{i},E_{j}\right)=\frac{\sum_{x\in E_{i},y\in E_{j}}S^{\prime }\left(x,y\right)\Delta x\Delta y}{|E_{i}\|E_{j}|},$$ (8)

where Δ*x* and Δ*y* are the average areas associated with each vertex on the respective spheres, that is, Δ*x* ≈ Δ*y* ≈ 8*π*/3, 675, assuming the vertices are uniformly distributed on Ω, and ∣*E*_*i*_∣ and ∣*E*_*j*_∣ denote the total area of parcels *E*_*i*_ and *E*_*j*_, respectively. We call $S_{A}^{\prime }$ the parcel-based SC matrix. The parcel-based SC matrix captures the idea that a parcellation of the brain reduces the complexity of SC data by merging the SC patterns of vertices.

#### Internal Evaluation Metrics.

We considered seven internal evaluation metrics: (a) approximation error, (b) 1-Wasserstein distance, (c) parcel homogeneity, (d) Calinski-Harabasz index, (e) proportion of contiguous parcels, (f) entropy, and (g) stability. Their definitions are given below.

##### Approximation error.

A parcellation merges the connectivity patterns of vertices by grouping them into parcels. This merging reduces the dimension of the high-resolution data at the cost of losing vertex-level connectivity information. Ideally, a parcellation should parcellate the high-resolution data in a way that minimizes this information loss. To quantify this loss, we created a metric called approximation error. Recall that $S_{A}^{\prime }$ is the parcel-based SC matrix derived from the parcellation A. Ideally, this parcel-based connectivity should resemble the connectivity patterns present in the full-resolution connectivity data in ${\bar{S}}^{\prime }$. However, a direct comparison between $S_{A}^{\prime }$ and ${\bar{S}}^{\prime }$ is difficult due to their differing dimensions: $S_{A}^{\prime }$ is a *K* × *K* matrix and ${\bar{S}}^{\prime }$ is a 3,675 × 3,675 matrix.

To overcome this, we create a new matrix denoted ${\bar{S}}_{A}^{\prime }$, which upscales the connectivity data in $S_{A}^{\prime }$ to match the dimension of ${\bar{S}}^{\prime }$. Suppose vertex *x* is assigned to parcel *E* and vertex *y* is assigned to parcel *E*^'^. Then, ${\bar{S}}_{A}^{\prime }\left(x,y\right)$, the connectivity between vertex *x* and *y* in the resolution-matched matrix, is given by $S_{A}^{\prime }\left(E,E^{\prime }\right)$. Additionally, we set the diagonal elements in ${\bar{S}}_{A}^{\prime }$ to zero to match the diagonal elements in ${\bar{S}}^{\prime }$. Finally, we define the approximation error, $AE\left(A\right)$, as follows:

$$AE\left(A\right)=\|{\bar{S}}^{\prime }-{\bar{S}}_{A}^{\prime }{\|}_{F},$$ (9)

where ‖ ⋅ ‖*_F_* denotes the Frobenius norm. Parcellations with a lower AE decrease the resolution of the data while preserving the connectivity patterns present in ${\bar{S}}^{\prime }$. Additionally, we computed AE at the subject level, providing a quantitative measure of how well a given parcellation preserves patterns in the subject-specific, high-resolution SC matrices.

##### 1-Wasserstein distance.

The distribution of connectivity values in ${\bar{S}}^{\prime }$ characterizes the variability of SC, and a parcellation should result in a resolution-matched matrix ${\bar{S}}_{A}^{\prime }$ that preserves this distribution. A common metric for quantifying the difference between two distributions is the Wasserstein distance. Given *P* and *Q* as probability measures on ℝ with cumulative distribution functions *F*(*x*) and *G*(*x*), respectively, the 1-Wasserstein distance *W*_1_ can be written as follows:

$$W_{1}\left(P,Q\right)=\left(\int_{0}^{1}\left|F^{-1}\left(t\right)-G^{-1}\left(t\right)\right|dt\right).$$ (10)

In our context, *P* and *Q* are probability measures associated with the connectivity distributions of ${\bar{S}}^{\prime }$ and ${\bar{S}}_{A}^{\prime }$, respectively, and *F* and *G* are their corresponding empirical cumulative distribution functions. We used the 1-Wasserstein distance to assess the similarity between the connectivity distributions of ${\bar{S}}^{\prime }$ and ${\bar{S}}_{A}^{\prime }$. A lower value of *W*_1_ indicates that the resolution-matched matrix preserves the connectivity distribution of the high-resolution SC data. The subject-level 1-Wasserstein distance was also computed to provide a quantitative measure of how well a given parcellation preserves the connectivity distribution of subject-specific, high-resolution SC matrices.

##### Parcel homogeneity.

Vertices within a parcel should have similar connections to other vertices; in other words, the SC profiles within a parcel should be homogeneous. To measure this, we followed Craddock et al. (2012) and computed the average Pearson’s correlation between every unique pair of vertex SC profiles within a parcel *E*_*k*_: $\bar{r}\left(E_{k}\right)=\sum_{x\neq y,x,y\in E_{k}}r({\bar{S}}^{\prime }(x,˙),{\bar{S}}^{\prime }(y,˙))/(\begin{array}{c} n_{i}\\ 2 \end{array}),$, where ${\bar{S}}^{\prime }(x,˙)$ and ${\bar{S}}^{\prime }(y,˙)$ represent the SC profiles of vertices *x* ∈ *E*_*k*_ and *y* ∈ *E*_*k*_, respectively, *n*_*i*_ is the total number of vertices in *E*_*k*_, and *r* denotes the Pearson’s correlation. “Parcel homogeneity” is then defined as the average of these values across all parcels in a parcellation A,

$$\text{Parcel Homogeneity}\left(A\right)=\frac{1}{K}\sum_{k=1}^{K}\bar{r}\left(E_{k}\right).$$ (11)

This parcel homogeneity metric takes values in [−1, 1]. Higher parcel homogeneity values indicate that the SC profiles of vertices within a parcel are similar, whereas lower values indicate that vertices within a parcel tend to have distinct connections to other vertices. Additionally, we computed parcel homogeneity at the subject level, by replacing ${\bar{S}}^{\prime }$ with *S*^'^, to measure how homogeneous within-parcel SC profiles are in subject-specific SC matrices.

##### The Calinski-Harabasz index.

While we expect vertices within a parcel to have homogeneous SC patterns, we also expect vertices in different parcels to have distinct SC patterns. To quantify this, we applied the Calinski-Harabasz index (CH), also known as the variance ratio criterion. Let *n*_*k*_ be the number of vertices in parcel *E*_*k*_, let *μ* be the 1 × 3,675 row-wise mean of ${\bar{S}}^{\prime }$, and let *μ_E_k__* be the 1 × 3,675 row-wise mean of the rows in ${\bar{S}}^{\prime }$ that correspond to the vertices in *k*-th parcel. Given a parcellation $A=\left\{E_{1},E_{2},\ldots ,E_{K}\right\}$, $CH\left(A\right)$ is calculated as follows:

$$CH\left(A\right)=\frac{\sum_{k=1}^{K}n_{k}{\left\|\mu_{E_{k}}-\mu \right\|}^{2}/\left(K-1\right)}{\sum_{k=1}^{K}\sum_{x\in E_{K}}{\left\|{\bar{S}}^{\prime }\left(x,.\right)-\mu_{E_{k}}\right\|}^{2}/\left(n-K\right)},$$ (12)

where ${\bar{S}}^{\prime }\left(x,.\right)$ is the SC profile of vertex *x*. The numerator of Equation 12 is the between-parcel sum of squares, and the denominator is the within-parcel sum of squares. Higher values of CH indicate that the parcellation A contains vertices that are more spread apart, in terms of their connectivity profile, between parcels than within parcels. We also computed the CH at the subject level.

##### Proportion of connected parcels.

The structural connectome is known to be spatially contiguous, that is, parcels should not be internally disconnected or split across the cortex. A parcellation with many disconnected parcels does not properly capture this property of the structural connectome. Given the adjacency matrices associated with the surface meshes introduced in the Image Data Processing and Connectome Extraction section (see Figure 2A), we also computed the proportion of connected parcels within a parcellation using a Depth-First Search (DFS) algorithm. For each parcel in the parcellation, the DFS algorithm starts at an arbitrary vertex within the parcel and iteratively explores its neighbors, via the surface mesh adjacency matrix. If every vertex within a given parcel can be reached from any starting vertex via DFS, then the parcel is considered connected. By performing this analysis, we can quantify the proportion of connected parcels in the parcellation. A higher value indicates that the parcellation contains a greater number of connected parcels.

##### Entropy of parcel sizes.

Parcellations with a highly nonuniform distribution of parcel sizes concentrate connectivity information unevenly across the cortical surface. Such parcellations may be restricted in capturing a wide variety of connectivity patterns. Entropy serves as a natural metric to quantify the uniformity of parcel sizes. Let *p*_*k*_ denote the proportion of vertices in parcel *k*. The entropy of a parcellation A is defined as follows:

$$H\left(A\right)=-\sum_{k=1}^{K}\frac{p_{k}\log_{2}\left(p_{k}\right)}{\log_{2}\left(M\right)},$$ (13)

where *M* is the total number of vertices being parcellated, *K* is total number of parcels, and log(*M*) serves as a normalizing factor. The entropy $H\left(A\right)$ ranges from 0 to 1. High values of *H* correspond to more uniformly distributed parcel sizes. This indicates that the parcellation is not dominated by large parcels and that the parcels are diverse, possibly capturing a wider variety of patterns in the data. Intuitively, the entropy of a parcellation is expected to increase as the number of parcels increases, albeit at the cost of increasing the complexity of the parcellation.

##### Adjusted mutual information.

We also used the adjusted mutual information (AMI) to compare the parcel assignments of two parcellations: $A=\left\{E_{1},E_{2},\ldots ,E_{K}\right\}$ and $\tilde{A}=\{{\tilde{E}}_{1},{\tilde{E}}_{2},\ldots ,{\tilde{E}}_{\tilde{K}}\}$, denoted as $AMI(A,\tilde{A}\}$. Let $p_{A}\left(E_{k}\right)$ and ${\tilde{p}}_{\tilde{A}}\left(E_{\tilde{k}}\right)$ be the proportion of vertices in parcel *E*_*k*_ of A and parcel $E_{\tilde{k}}$ of $\tilde{A}$, respectively, and let $p_{A\tilde{A}}(E_{k},E_{\tilde{k}})$ be the proportion of vertices common to parcels *E*_*k*_ and $E_{\tilde{k}}$. Then, the MI is given by the following.

$$MI\left(A,\tilde{A}\right)=\sum_{k}^{K}\sum_{\tilde{k}}^{\tilde{K}}p_{A\tilde{A}}\left(E_{k},E_{\tilde{k}}\right)\log\frac{p_{A\tilde{A}}\left(E_{k},E_{\tilde{k}}\right)}{p_{A}\left(E_{k}\right)p_{\tilde{A}}\left(E_{\tilde{k}}\right)}.$$

There are potentially two problems with this form: (a) Two completely randomized parcellations may have nonzero MI values, and (b) $MI(A,\tilde{A})$ tends to increase as the number of parcels in A or $\tilde{A}$ increases. For these reasons, it is popular to use a generalized hypergeometric model of randomness, that is, the two parcellations are assumed to have been chosen at random with the constraint that they retain their number of parcels and their number of vertices within each parcel (Hubert & Arabie, 1985). Under this model, the expected MI $E\left(MI\right)$ can be derived (Vinh, Epps, & Bailey, 2009). To adjust $MI(A,\tilde{A})$, we follow Hubert and Arabie (1985) and Vinh et al. (2010):

$$AMI\left(A,\tilde{A}\right)=\frac{MI\left(A,\tilde{A}\right)-E\left(MI\left(A,\tilde{A}\right)\right)}{\max\left\{H\left(A\right),H\left(\tilde{A}\right)\right\}-E\left(MI\left(A,\tilde{A}\right)\right)},$$ (14)

where $H\left(A\right)$ is defined in Equation 13. If A and $\tilde{A}$ are identical, then the AMI value is 1. If the MI between A and $\tilde{A}$ equals the expected MI due to chance alone, then the AMI will be 0. We consider two parcellations to be similar if they have a high AMI and dissimilar if their AMI is low.

##### Stability.

A method for parcellating the brain should be stable across homogeneous groups of subjects. To quantify the stability of CoCoNest, we randomly created 10 batches of 100 subjects from the 897 subject considered. Each of these batches was then fed into the CoCoNest pipeline (see Figure 1) to create 10 nested families of parcellations. We then used the AMI, as detailed above, to quantify how similar these nested families were to each other and to the CoCoNest family constructed using all 897 subjects.

#### External Evaluation Metrics.

We use the following two external evaluation metrics to assess parcellation performance.

##### Trait prediction.

An important task in connectome studies is to understand the relationship between brain connectivity and various demographic, behavioral, or physiological traits (Gosnell et al., 2019; Hirsiger et al., 2016; Smith, 2016; Zimmermann, Griffiths, & McIntosh, 2018). A derived brain parcellation should facilitate such analysis tasks. To evaluate this, we predicted selected behavioral traits available from the HCP beginning with different parcellations. These traits included score on reading recognition test (reading) number of correct responses on the Penn Matrix test (fluid intelligence), number of correct responses on the Penn Line Orientation test (spatial orientation), number of correct responses on the Penn Word Memory test (verbal episodic memory), score on the openness to experiences section of the Five Factor Model (openness), and gender. The variable names in parenthesis follow the convention of Ooi et al. (2022). Since our primary goal was to compare all parcellations under a straightforward and efficient predictive model, rather than identifying the optimal model for linking brain connectivity to human traits, we opted to use three simple models: principal component regression (PCR), ridge regression, and support vector regression (SVR) with a linear kernel. For PCR, a ridge regression model was used to predict *y* with the derived PC scores.

In our experiments, for each parcellation, each subject’s high-resolution SC matrix was converted into a parcel-based SC matrix. The log transformation was omitted to achieve better prediction results, and thus, *S*(*x*, *y*) replaced *S*′(*x*, *y*) in Equation 8. We followed the standard convention and set self-connections in the parcel-based SC matrix to 0. The parcel-based SC matrix for each subject was then vectorized to derive a *N* × *K*(*K* − 1)/2 matrix, where *N* is the number of subjects and *K* is the number of parcels in the considered parcellation. To predict a selected trait *y*, the data were randomly split 50 times into training (80%) and testing (20%) sets. In each split, each of the three considered models were trained, with their hyperparameters (e.g., the number of PC scores to retain for PCR or the penalty parameter in ridge) chosen using fivefold cross-validation. Subsequently, the testing set was fed into the trained model to predict the values of the response *y*. The model’s performance was evaluated using the Pearson correlation between the measured and predicted *y* values. The performance of a parcellation across the 50 data splits is summarized by the mean and the standard error of the mean of the correlations. To assess a parcellation’s performance in predicting gender, we used a logistic regression (LR) model with PC scores (PCLR), *L*_2_-regularized LR, and a linear support vector classifier (SVC). The performance was measured using the AUC area under the receiver operating characteristic (ROC) curve.

A parcellation that is well-suited for exploring the relationship between brain connectivity and subject-level traits should have a strong predictive power for those traits.

##### Test–retest identifiability.

SC has been shown to be highly reproducible (Bonilha et al., 2015; Prčkovska et al., 2016; Zhang et al., 2018), that is, SCs generated by the same individual are notably more similar than SCs derived from different individuals. We draw on a metric, known as neural identifiability (${NI}_{A}$), introduced by Mansour L., Tian, Yeo, Cropley, and Zalesky (2021) to assess how well a parcellation preserves this inherent reproducibility of SC. To define NI, we made use of the imaging from 897 subjects from the HCP, where 37 of these subjects underwent an additional imaging session at a later date. We designate the SC data derived from the initial scans from the 897 subjects as the test dataset and the SC data derived from the subsequent second scans from the subset of 37 subjects as the retest dataset. To calculate ${NI}_{A}$ for a given parcellation A, we first constructed a parcel-based SC matrix, using Equation 8, from each of the full-resolution test and retest datasets.

Let $S_{A}^{\prime \left(i\right)}$ denote the parcel-based SC matrix of subject *i*. We defined the similarity $R_{A}\left(i,j\right)$ between the parcel-based SC matrices of subject *i* and *j* as the average correlation coefficient between the corresponding rows of both matrices.

$$R_{A}\left(i,j\right)=\frac{1}{K}\sum_{k=1}^{K}\text{corr}(S_{A}^{\prime \left(i\right)}\left(E_{k},.\right),S_{A}^{\prime \left(j\right)}\left(E_{k},.\right).$$ (15)

We then averaged the 37 intrasubject and 37 × 897 intersubject similarities, denoted ${\bar{R}}_{A}^{\text{intra}}$ and ${\bar{R}}_{A}^{\text{inter}}$, respectively. The Cohen’s *D* effect size was then used to define ${NI}_{A}$.

$${NI}_{A}=\frac{|{\bar{R}}_{A}^{\text{intra}}-{\bar{R}}_{A}^{\text{inter}}|}{s},$$ (16)

where *s* is the pooled standard deviation of the similarities. A parcellation that achieves a higher NI score better separates the intrasubject from the intersubject similarities, and thus parcellates the structural connectome more reliably. Such a parcellation creates a distinguishable SC “fingerprint” for an individual and effectively reflects the high reproducibility of SC.

##### Validation on an external dataset.

A good parcellation of the structural connectome should capture meaningful SC patterns across diverse populations. To assess this, we evaluated the performance of CoCoNest on the ABCD study dataset (Casey et al., 2018).

To do this, we first downloaded dMRI and sMRI data from 493 subjects who participated in the ABCD study. Details on the minimal processing pipelines and acquision protocols can be found in Casey et al. (2018) and Hagler et al. (2019). To avoid interscanner effects, we only considered subjects who were scanned using a Siemens manufactured scanner. The downloaded ABCD imaging data were then processed through the SBCI Pipeline (see the Image Data Processing and Connectome Extraction section). As before, the SBCI pipeline performed standard preprocessing steps and output a high-resolution SC matrix for each subject based on a continuous representation of SC.

The performance of a parcellation was then measured using a collection of the introduced internal evaluation metrics introduced in the Quantifying Parcellation Performance section, namely, approximation error, 1-Wasserstein distance, the Calinski-Harabasz index, and parcel homogeneity. For external evaluation, we predicted selected behavioral traits available from the ABCD study. These traits included vocabulary, fluid intelligence, fluid cognition, crystallized cognition, reward responsiveness, and gender. These variable names follow the convention of Ooi et al. (2022).

## RESULTS

We used high-quality imaging data from the HCP to construct a multiresolution family of CoCoNest parcellations based on structural connectome data.

Figure 1E shows the subtrees corresponding to three members of the CoCoNest family with 9, 68, and 360 terminal nodes. Figure 1F displays the parcellations derived from these subtrees along with the corresponding parcel-based representation of ${\bar{S}}^{\prime }$, derived using Equation 8.

We used the internal and external evaluation metrics described above to evaluate the performance of members of the CoCoNest family, and to compare CoCoNest with the widely used parcellations in the literature, including the anatomical-based Desikan (Desikan et al., 2006) and Destrieux (Destrieux et al., 2010) parcellations; the structural connectome-based Brainnetome parcellation (Fan et al., 2016); the functional connectome-based Yeo-17 (Yeo et al., 2011), Gordon (Gordon et al., 2016), and multiresolution Schaefer (Schaefer et al., 2018) parcellations; and the multimodal Glasser parcellation (Glasser et al., 2016).

### Internal Evaluation Results

**Figure 4.**
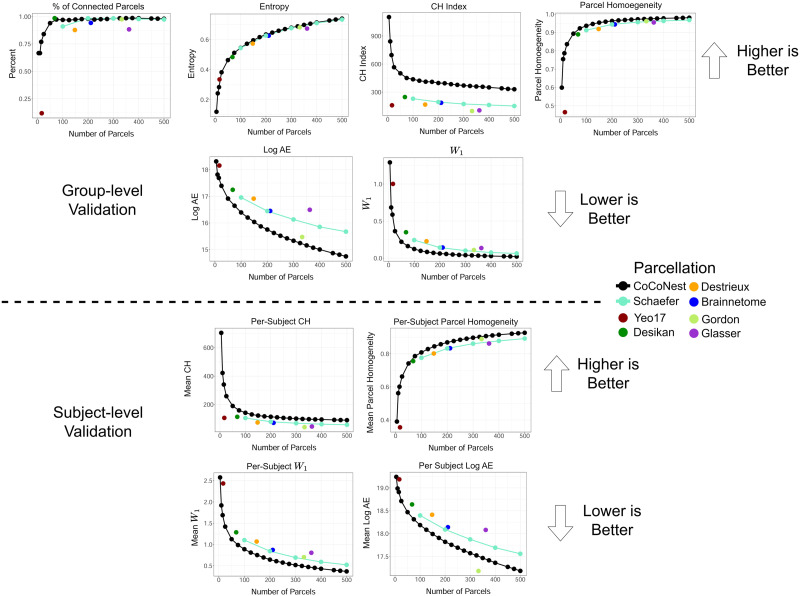
Results of the internal evaluation metrics for assessing parcellation performance. The top panel shows the internal validation performed on the average connectivity matrix, ${\bar{S}}^{\prime }$, while the bottom panel shows the validation performed on the subject-specific connectivity data. For these metrics, members of the CoCoNest family are competitive with, and often superior to, widely used parcellations in the literature. In the figure, “CH” denotes the Calinski-Harabasz index, “AE” denotes the approximation error, and “*W*_1_” denotes the 1-Wasserstein distance.

The comparison results for the internal evaluation metrics are shown in Figure 4. Higher values in the proportion of contiguous parcels, entropy, and CH index indicate better performance; these are shown in the top panel. Conversely, lower values of *W*_1_ and logAE indicate better performance; these are shown in the bottom panel.

The results show that members of the CoCoNest family containing more than 100 parcels possess several desirable characteristics. Firstly, they feature homogeneous, uniformly sized, and connected parcels, as indicated by the proportion of connected parcels and the entropy of parcels. Secondly, these parcellations preserve high-resolution SC patterns in both ${\bar{S}}^{\prime }$ and subject-specific high-resolution SC data, as seen in the log AE measure. Thirdly, they have a much higher ratio of between-parcel variation to within-parcel variation (as measured by CH) compared with the other parcellations considered. Lastly, CoCoNest members outperform the other parcellations with similar sizes in both the mean per-subject AE and the mean per-subject *W*_1_ metrics, indicating that CoCoNest members better preserve high-resolution SC patterns in subject-specific data.

We found small deviations from connectedness in several parcels in the considered parcellations from the literature (e.g., a single parcel in the Desikan parcellation). These deviations are a result of small errors incurred during downsampling from the high-resolution white matter surfaces (see the Image Data Processing and Connectome Extraction section). Examples of such small deviations are given in the Examples of Non-connected Clusters section of the Supporting Information.

**Figure 5.**
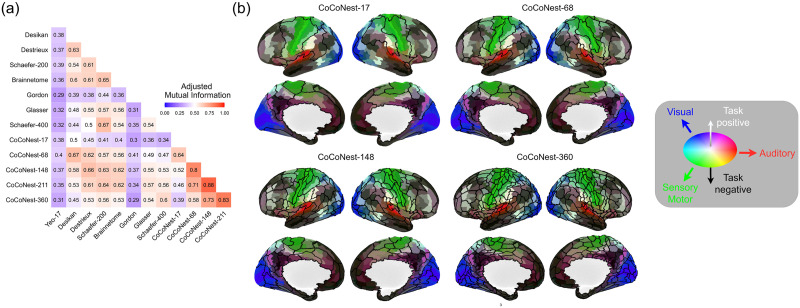
(A) The AMI between popular parcellations in the literature and the CoCoNest family member with a similar number of parcels. (B) CoCoNest members (outlined in black) overlaid on top of the Glasser parcellation (in color). The color scale of the Glasser parcellation follows Glasser et al. (2016).

Figure 5A shows the AMI between selected members of the CoCoNest family and other parcellations with a similar number of parcels. The Yeo-17 and Gordon parcellations show the most dissimilarity to the other parcellations, which is expected since they were constructed using fMRI data. We found moderate similarity (AMI ≈ 0.60) between CoCoNest family members and the Desikan, Destrieux, Brainnetome, Glasser, and Shaefer parcellations. Figure 5B overlays members of the CoCoNest family (outlined in black) on top of the Glasser parcellation. The color scheme of the Glasser parcellation allowed us to visualize how parcels from the CoCoNest family overlap with known anatomical and functional regions. For example, the CoCoNest member with 17 parcels contains large parcels covering the visual and sensorimotor regions of the brain. As the number of parcels increase, CoCoNest members are characterized by elongated parcels along the sensorimotor regions of the brain, similar to the Glasser parcellation, and larger parcels in the frontal lobe. Additionally, members of the CoCoNest family show a high degree of symmetry, with parcels on the left hemisphere mirroring the shape and size of similarly located parcels on the right hemisphere.

**Figure 6.**
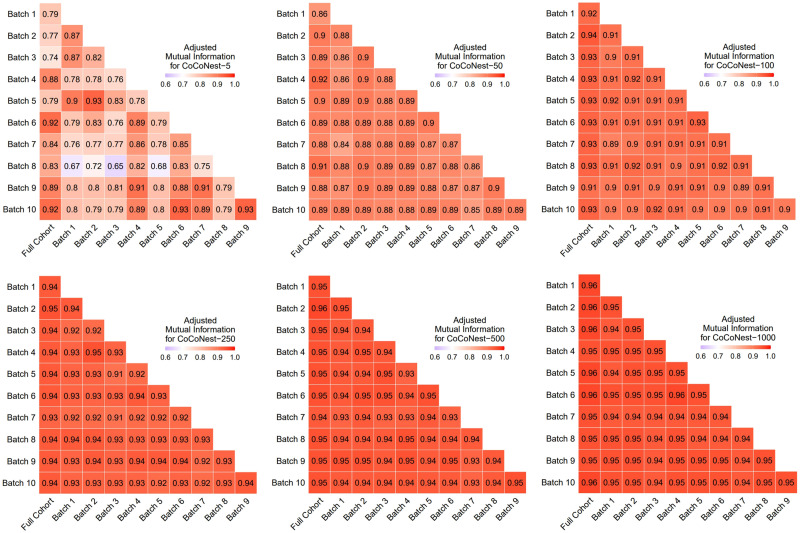
Color map showing the AMI between members of the CoCoNest family derived from 10 batches of 100 subjects using the CoCoNest parcellation pipeline. Each of these parcellations was also compared to the CoCoNest member constructed from the full set of 897 subjects. Higher AMI values indicate greater similarity between the parcellations.

Figure 6 shows the stability of the CoCoNest procedure, as measured by the AMI between members of CoCoNest families derived from different subsets of the 897 subjects. Here, we include results for CoCoNest-5, CoCoNest-50, CoCoNest-100, CoCoNest-250, and CoCoNest-1000. The parcellations were remarkably similar, with the AMI value exceeding 0.9 for every parcellation size greater than 50 parcels. These results show that CoCoNest parcellations are robust and stable across homogeneous groups of subjects.

### External Evaluation Results

**Figure 7.**
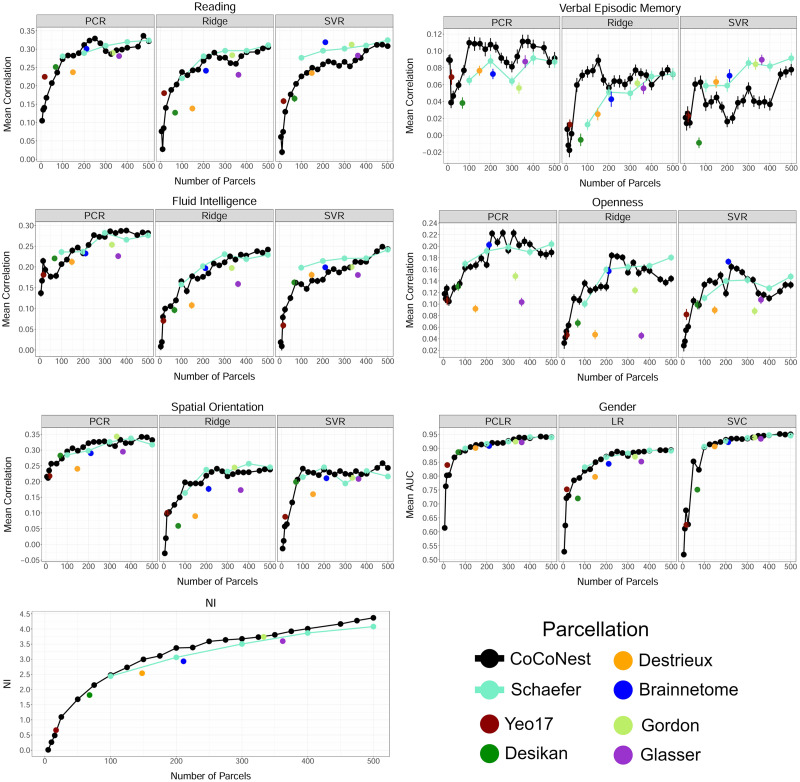
Results of predicting different human traits and neural identification using parcel-based SC matrices generated by different parcellations. For predicting human traits, the mean predictive performance (correlation between predicted and reported values) over 50 training/testing splits is plotted versus the number of parcels in each parcellation. The error bars represent the standard error of the mean.

Figure 7 shows the trait prediction and neural identification performance of members of the CoCoNest family and the other parcellations considered. With a similar number of parcels, CoCoNest family members consistently outperformed the other parcellations considered across all prediction models. Additionally, after 250 parcels, the performance of CoCoNest members tends to plateau. From the NI metric, we found that members of the CoCoNest family were competitive in reflecting the high reproducibility of SC, and thus creating a more distinguishable SC fingerprint for an individual. It is interesting to note that although the Yeo-17 parcellation was constructed using functional connectome data, it provides an SC fingerprint competitive with CoCoNest members and the Desikan atlas.

### Validation on the ABCD Dataset

**Figure 8.**
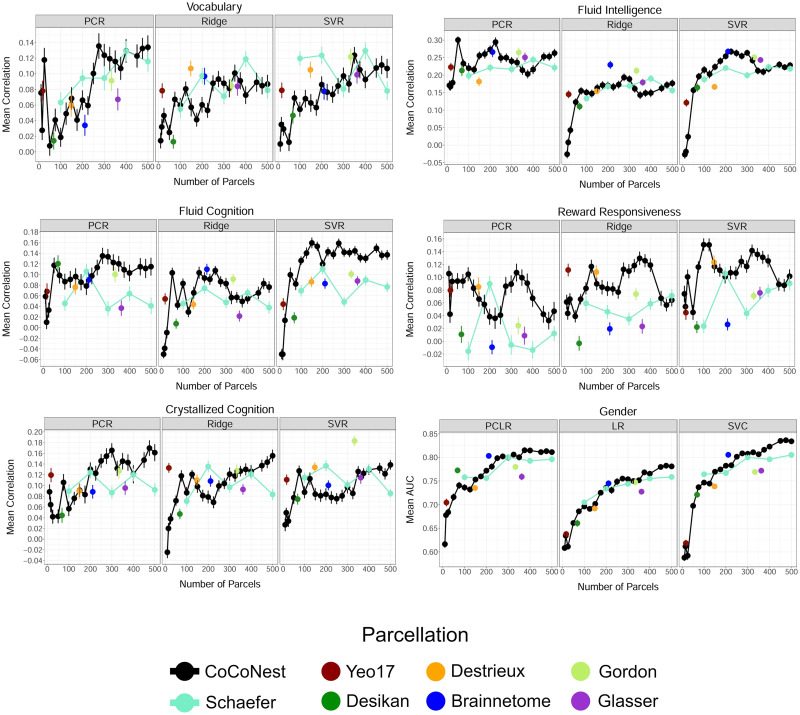
Trait prediction results using the ABCD dataset for each parcellation. The mean predictive performance (correlation between predicted and reported values) over 50 training/testing splits is plotted versus the number of parcels in each parcellation. The error bars represent the standard error of the mean.

Figure 8 shows the trait prediction results using data downloaded from the ABCD study. Echoing the results of the HCP external validation, we found that members of the CoCoNest family were simultaneously competitive with the other parcellations considered and often showed superior performance across all prediction models. Additionally, as in the HCP external validation, we found that the CoCoNest member with 250 parcels consistently achieved the best, or close to the best, performance across the considered resolutions. All parcellations considered were competitive in the internal evaluation metrics, with CoCoNest achieving marginal improvements in performance. These internal validation results can be found in Supporting Information Section S1.

Additionally, a natural question is whether a family of CoCoNest parcellations derived from the ABCD data, referred to as CoCoNest_ABCD, outperforms the proposed CoCoNest family, derived from the HCP data, when validated using the ABCD data. Our findings indicate that CoCoNest_ABCD parcellations indeed show moderately better performance across both internal and external evaluation metrics. These results are reported in Supporting Information Section S1.2.

### CoCoNest as an Exploratory Framework

**Figure 9.**
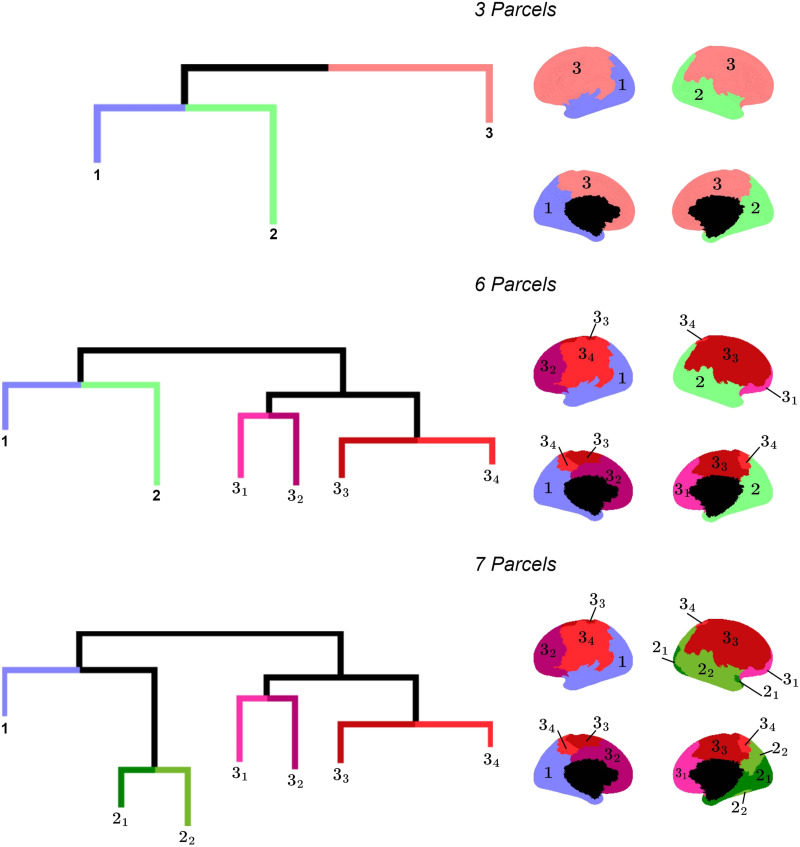
Visualization of the final stages of the error-complexity pruning procedure. Colors and numbers represent distinct parcels on the brain’s surface. Numbers with a subscript indicate a parent–child relationship between parcels. CoCoNest aims to group brain regions based on similar structural connectivity patterns and separates those with differing patterns. The last parcellation before reaching the root node contains three parcels: it parcellates the frontal lobe of both hemispheres together, while separating the temporal and occipital lobes of the left hemisphere from those on the right. These structural connectivity patterns may give rise to the similar and distinct functional roles of these brain regions.

The nested structure of the parcellations in the CoCoNest family can provide a valuable framework for exploratory analyses into the organization of the structural connectome. To give an example, consider the final stages of the error-complexity pruning procedure shown in Figure 9. Notably, CoCoNest does not separately partition the left and right hemispheres as might be expected. Rather, it merges the frontal and parietal lobes of both hemispheres into one parcel (Parcel 3 in Figure 9), while merging the temporal and occipital lobes of the left hemisphere separately from those on the right hemisphere (Parcels 1 and 2 in Figure 9). This approach suggests that the tree creation and pruning procedures identify similar SC patterns in the frontal and parietal lobes across both hemispheres, but disparate patterns in the occipital and temporal lobes. This division of lobes is consistent with existing literature that highlights functional differences between the left and right temporal lobes (Chan et al., 2009; Scott, Blank, Rosen, & Wise, 2000). As this example illustrates, by navigating the family of CoCoNest parcellations, researchers can gain a better understanding of the structural variations between brain regions and can investigate how these variations might correlate with functional roles.

**Figure 10.**
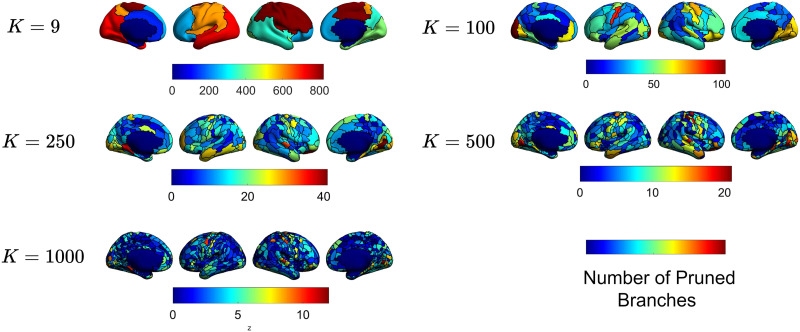
A visualization of the number of branches pruned from the full CoCoNest tree to arrive at the parcels in various CoCoNest members.

The error-complexity pruning algorithm used to construct the CoCoNest family aims to strike a balance between the complexity of a parcellation and how well it represents the high-resolution SC data ${\bar{S}}^{\prime }$. For each terminal node in a subtree corresponding to a CoCoNest member, we calculated the number of branches pruned from the full CoCoNest tree to arrive at that node. A large number of branches pruned in a brain region suggests that many splits were eliminated. This elimination is done to balance complexity with accuracy. In other words, the higher the number of pruned branches in a region, the less distinct the SC profiles in that region are. Figure 10 shows this calculation for members of the CoCoNest family with roughly 10, 100, 250, 500, and 1,000 parcels.

We observe that, across various resolutions, the CoCoNest pruning procedure favors pruning parcels in regions including the occipital, precentral, postcentral, and temporal lobes. This suggests that combining the SC patterns in these lobes reduces the complexity of the connectome data while still preserving high-resolution SC patterns. When conducting SC analyses using the CoCoNest family, researchers can use the number of pruned branches below a parcel to inform hypotheses about which parcels possess crucial SC patterns.

As mentioned earlier, a parcellation serves as a natural bridge between neuroscience and network science. By treating the parcels as nodes and the structural connections as edges, $S_{A}$ can be viewed as a weighted network with *K* nodes. In order to showcase how members of the CoCoNest family can be used as an exploratory tool in network-based analyses, we used them to investigate the rich club effect in the structural connectome. To carry out a more interpretable analysis, the log transformation introduced in the Image Data Processing and Connectome Extraction section was omitted for this task.

Recall that the degree of a node in a network is measured by the number of outgoing connections that it has to other nodes. Similarly, the weighted degree of a node is the sum of the weights of these outgoing connections. Rich clubs in networks are high degree nodes that are more strongly connected to each other than nodes of lower degrees. Previous work has shown that the structural connectome strongly displays the rich club effect (Grayson et al., 2014; Liu et al., 2021; van den Heuvel, Kahn, Goñi, & Sporns, 2012; van den Heuvel & Sporns, 2011). We follow (van den Heuvel & Sporns, 2011) and use the normalized, weighted rich club coefficient to probe for the rich club effect in the structural connectome. A normalized rich club coefficient greater than 1 for successive values of *k* indicates the presence of the rich club effect.

**Figure 11.**
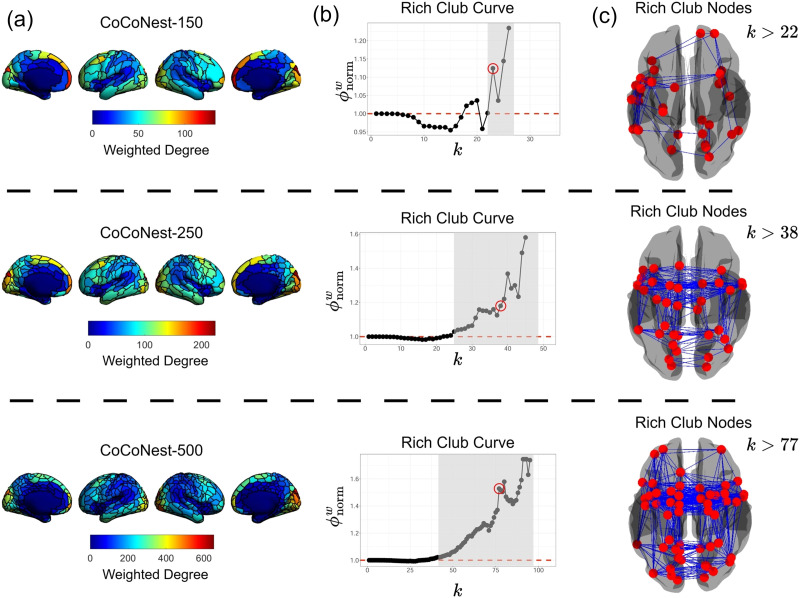
A multiscale network analysis with three members of the CoCoNest Family (CoCoNest-150, CoCoNest-250, and CoCoNest-500). (A) The weighted degree of each parcel on the cortical surface. Parcels in the frontal and parietal lobes were characterized by lower weighted degrees, whereas parcels in the occipital lobe showed higher weighted degrees. (B) The rich club curve. The region shaded in gray indicates the rich club regime where $\phi_{\text{norm}}^{w}$ remains greater than one for successive values of *k*. Each member of the three CoCoNest family members displayed evidence of the rich club effect. (C) The rich club nodes on the cortical surface (represented as red balls), along with the connections between them (represented as blue line segments). Rich club nodes across these three members include parcels that overlap with the superior frontal cortex, somatosensory regions, and the inferior parietal lobe across both hemispheres.

Figure 11 shows the weighted degrees of each parcel on the cortical surface, the normalized rich club coefficients, and the identified rich club nodes for networks derived from CoCoNest-150, CoCoNest-250, and CoCoNest-500. In general, we found that parcels in the frontal and parietal lobes showed lower weighted degrees, whereas parcels in the occipital lobe showed higher weighted degrees. For all three members, we found evidence of the rich club effect. Notably, the rich club nodes across these members include parcels that overlap with the superior frontal cortex, somatosensory regions, and the inferior parietal lobe across both hemispheres. These findings are similar to previous work (Grayson et al., 2014; Liu et al., 2021; van den Heuvel et al., 2012; van den Heuvel & Sporns, 2011). We also found similar results using the other considered external parcellations (see Supporting Information Section S3). Additionally, we found that nodes in the anterior portion of the frontal lobe and the posterior portion of the occipital lobe showed variable rich club status across the three resolutions. For instance, while no rich club nodes were found in the anterior portion of the frontal lobe while analyzing the network created by CoCoNest-250, rich club nodes were present in these areas when analyzing the networks created by CoCoNest-150 and CoCoNest-250. Further details on this rich club analysis can be found in Supporting Information Section S3. These observations highlight how network-based insights into the structural connectome can vary with resolution, demonstrating CoCoNest’s usefulness for conducting tractable, multiscale analyses of the structural connectome.

### Selection and Sharing of CoCoNest

**Figure 12.**
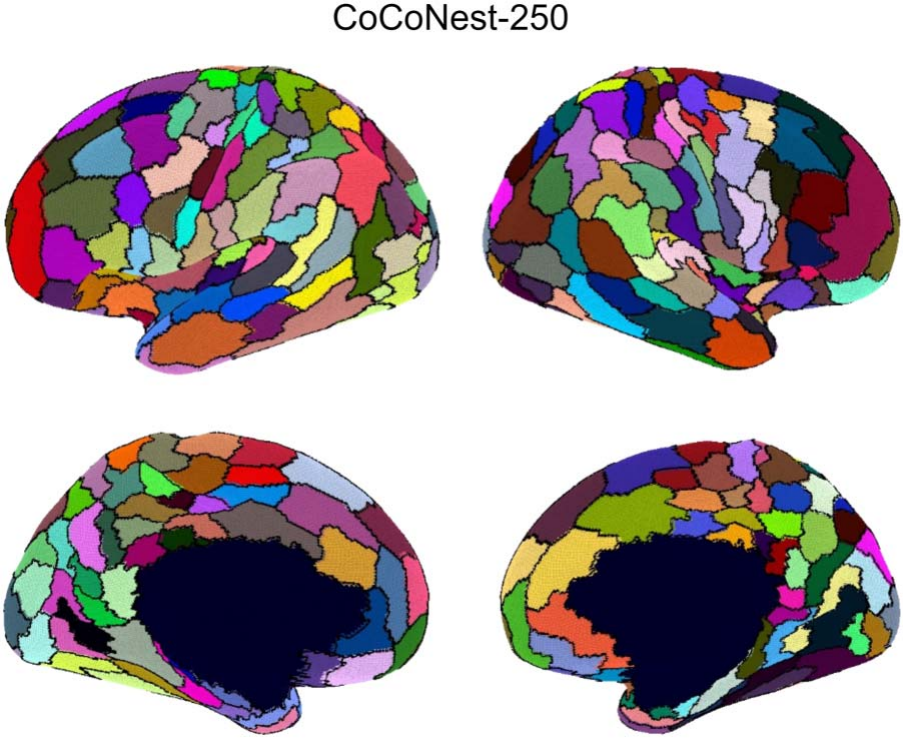
CoCoNest-250, the member of the CoCoNest family with 250 parcels. We found that CoCoNest-250 showed competitive performance, not only among other members of the CoCoNest family but also compared with other widely used parcellations in the literature. For researchers seeking a single-resolution parcellation, we recommend CoCoNest-250 for its competitive performance.

Since each member of the CoCoNest family corresponds to a unique subtree within the full CoCoNest tree, the CoCoNest family enables researchers to explore the multiresolution nature of the structural connectome. While the hierarchical structure may offer enhanced insights into brain organization, researchers may be interested in using just a single resolution, or a CoCoNest member, for their analyses. According to the internal and external validation results in Figures 4 and 7, larger CoCoNest parcellations tend to show diminishing improvements in performance after about 250 parcels. Therefore, if one is seeking a single-resolution parcellation for the HCP data considered above, a natural candidate is CoCoNest-250 (shown in Figure 12) which performs well on both the internal and external evaluation metrics.

Every member of the CoCoNest family can be freely accessed at https://github.com/sbci-brain/CoCoNest. Additionally, the repository contains scripts to convert any member of the CoCoNest family to the fsLR-32 k, fsaverage, and MNI152 template spaces. The repository also contains the scripts used to create the CoCoNest family from SBCI-processed brain connectivity data.

## DISCUSSION

In this work, we leveraged recent advances in structural connectome reconstruction, as well existing techniques from hierarchical clustering and error-complexity pruning, to construct a nested, multiresolution family of parcellations, called the CoCoNest family. The CoCoNest family provides researchers with insights into the structural connectome across multiple resolutions. The multiresolution nature of the CoCoNest family is naturally aligned with the current consensus in the literature on the multiscale nature of the brain. Through an extensive battery of internal and external evaluation metrics, we have shown that the CoCoNest family is simultaneously competitive with widely used parcellations in the literature, including the Yeo-17, Desikan, Destrieux, Brainnetome, Gordon, Schaefer, and Glasser parcellations (Desikan et al., 2006; Destrieux et al., 2010; Fan et al., 2016; Glasser et al., 2016; Gordon et al., 2016; Schaefer et al., 2018; Yeo et al., 2011). In particular, CoCoNest members with a similar number of parcels as these widely used parcellations often show superior performance in a number of unsupervised and predictive metrics.

Additionally, there are several alternative methods for creating a connectome-based parcellation. These methods can be broadly categorized into gradient-based methods (Cohen et al., 2008; Gordon et al., 2016; Schaefer et al., 2018; Wig et al., 2014), which identify abrupt changes in connectivity across the cortical surface, and statistical clustering methods, which cluster regions based on similar connectivity patterns. Widely used statistical clustering methods include mixture models (Baldassano, Beck, & Fei-Fei, 2015; Golland, Golland, & Malach, 2007; Kong et al., 2019; Lashkari et al., 2012; Moyer, Gutman, Jahanshad, & Thompson, 2017; Roca et al., 2010; Yeo et al., 2011), k-means clustering (Flandin et al., 2002; Kahnt, Chang, Park, Heinzle, & Haynes, 2012; Salehi, Karbasi, Shen, Scheinost, & Constable, 2018), and spectral clustering (Chen et al., 2013; Craddock et al., 2012; Fan et al., 2016; Thirion, Varoquaux, Dohmatob, & Poline, 2014). These methods typically produce a single parcellation with a predefined number of parcels. However, recent work has revealed the multiscale nature of human brain connectivity, where the interactions and distinctions between scales are thought to be critical to the overall functionality of the brain (Bassett & Siebenhühner, 2013; Betzel & Bassett, 2017; Meunier, Lambiotte, & Bullmore, 2010; R. Wang et al., 2019). As opposed to generating a sequence of predefined number of parcels and using the aforementioned methods, hierarchical clustering approaches, like the proposed CoCoNest, have been used to better represent the multiscale nature of the connectome (Blumensath et al., 2013; Cammoun et al., 2012; Diez et al., 2015; Eickhoff et al., 2011; Gallardo, Wells, Deriche, & Wassermann, 2018; Kurmukov et al., 2020; Michel et al., 2012; Moreno-Dominguez, Anwander, & Knösche, 2014). This approach represents parcels as being composed of smaller subparcels, thus capturing both the interactions and distinctions between scales. Here, we call this spatial, multiscale representation of the connectome a multiresolution representation to highlight its nested structure and to distinguish it from the temporal and topological scales of brain networks often studied (Betzel & Bassett, 2017).

The main decisions in these hierarchical parcellation methods center on creating a hierarchical structure from connectome data and deriving parcellations from this structure. A simple approach is to iteratively merge nearby vertices in high-resolution structural connectome data to form a hierarchical structure (Cammoun et al., 2012). However, this method does not allow the structural connectome data itself to directly influence the resulting hierarchical structure. Modularity maximization algorithms, particularly the Louvain algorithm, have also been commonly used to create hierarchical structures from structural connectome data (Diez et al., 2015; Kurmukov et al., 2020). Yet, this approach has been shown to be limited in capturing partitions with small clusters, especially when used with dense, high-resolution data (Fortunato & Barthélemy, 2007). Additionally, implementations of hierarchical clustering for structural connectome data predominantly focus on subject-level parcellations (Kurmukov et al., 2020; Moreno-Dominguez et al., 2014). However, aggregating individual hierarchical structures or computing a consensus hierarchy is challenging and often avoided, making group-level analysis of the organization of the structural connectome difficult. Finally, many existing parcellations have not undergone rigorous validation by both internal and external metrics across independent datasets. In this work, we have shown that parcellations derived using the CoCoNest framework effectively address the above limitations.

Despite the advantages of the CoCoNest framework, there are several limitations. First, CoCoNest relies solely on the density of white matter fiber tracts between brain regions. In order to fully characterize the structural connectome, it may be important to include additional information about the SC between vertices. For example, considering the shape and curvature of these tracts could offer a more nuanced characterization of each connection. Second, CoCoNest is built on the population-averaged high-resolution *SC* matrix. Such averaging may distort SC characteristics found at the subject level. Exploring more refined methods to construct a consensus SC matrix for a population might enhance the ability of CoCoNest to characterize the structural connectome. Similarly, incorporating measures of uncertainty arising from the variability of SC may yield a more rigorous notion of similarity between SC patterns. Lastly, CoCoNest is completely deterministic, which restricts our ability to study the error in the CoCoNest tree or explore alternative trees that were likely to be generated from the data (Peixoto, 2023). Leveraging tools from the literature on hierarchical random graphs (Briercliffe, 2023) or nested stochastic block models (Peixoto, 2014) may provide avenues for further analyses and facilitate statistical inference on nested parcellations like the CoCoNest family.

As the CoCoNest family was constructed using dMRI data, it is important to note that numerous challenges exist in accurately uncovering the underlying white matter architecture, which could impact any subsequent analysis, including our proposed CoCoNest family. Tractography algorithms strive to infer global connectivity from local diffusion information. This approach will inevitably produce false-positive and omit true-positive fiber tracts (Maier-Hein et al., 2017; Rheault, Poulin, Caron, St-Onge, & Descoteaux, 2020). Additionally, the complex geometry of these tracts may produce diffusion signals that generate biases in tractography. For example, overlapping fiber bundles with independent endpoints have been shown to produce invalid structural connections (Maier-Hein et al., 2017) (also called the bottleneck effect; Rheault et al., 2020). Moreover, the reconstructed fiber tracts can have arbitrary endpoints inside of the brain rather than on the cerebral cortex, the number of short-range fiber tracts may be overestimated, and significant gyral bias can occur (Reveley et al., 2015; Rheault et al., 2020; St-Onge et al., 2018). To mitigate the influence of these biases on the CoCoNest family, we have used high-quality imaging data from the HCP and SET, which has been shown to reduce such biases. However, we acknowledge that our method is not entirely immune to these limitations. As brain imaging technology and tractography algorithms evolve, it is important to reevaluate parcellations derived from any method of structural connectome reconstruction.

We have made all members of the CoCoNest family freely available, along with the code used to construct the entire family, at https://github.com/sbci-brain/CoCoNest. In general, the methodology presented in this work can be used to create a “CoCoNest”-like parcellation from any SC matrix. The choice of similarity and linkage functions and the error-complexity objective function are flexible. However, we believe our method works best with dense, high-resolution SC matrices (as produced from the SBCI pipeline; Cole et al., 2021).

## ACKNOWLEDGMENTS

Data were provided by the Human Connectome Project, WU-Minn Consortium (Principal Investigators: David Van Essen and Kamil Ugurbil; 1U54MH091657) funded by the 16 NIH Institutes and Centers that support the NIH Blueprint for Neuroscience Research; and by the McDonnell Center for Systems Neuroscience at Washington University. Nobel’s research was supported by NSF Grant DMS 2113676. Zhang gratefully acknowledges support from the Ralph E. Powe Junior Faculty Enhancement Award and NIH Grant R25DA058940.

## SUPPORTING INFORMATION

Supporting information for this article is available at https://doi.org/10.1162/netn_a_00409.

## AUTHOR CONTRIBUTIONS

Adrian Allen: Conceptualization; Data curation; Formal analysis; Investigation; Methodology; Software; Validation; Visualization; Writing – original draft; Writing – review & editing. Zhengwu Zhang: Conceptualization; Data curation; Formal analysis; Funding acquisition; Investigation; Methodology; Project administration; Resources; Software; Supervision; Validation; Writing – original draft; Writing – review & editing. Andrew Nobel: Conceptualization; Formal analysis; Investigation; Methodology; Project administration; Resources; Supervision; Validation; Writing – original draft; Writing – review & editing.

## DATA AVAILABILITY STATEMENT

Data and code are available at https://github.com/sbci-brain. This repository contains codes to extract members of the CoCoNest family and project them to popular template spaces.

## Supplementary Material



## TECHNICAL TERMS

Parcellation: A partitioning of the brain into discrete regions of interest called parcels.
Structural connectome: A comprehensive map of interconnecting white matter fiber tracts between brain regions.
Diffusion MRI (dMRI): An imaging technique that maps the diffusion of water molecules in biological tissue.
Tractography: A method that leverages dMRI data to reconstruct white matter fiber tracts.
Continuous representation of structural connectivity: A representation of structural connectivity as a continuous function across the brain’s surface.
Full binary tree: A hierarchical, tree-based data structure in which each node has either zero or two children nodes.
Internal parcellation evaluation: A method for evaluating a parcellation based solely on the intrinsic properties of the parcels.
External parcellation evaluation: A method for evaluating a parcellation using auxiliary or external information.
Tree pruning: A technique for removing branches from a tree data structure.
