# Supplementary material for: CoCoNest: A continuous structural connectivity-based nested family of parcellations of the human cerebral cortex
Source: Netw Neurosci. 2024 Dec 10;8(4):1439–66. doi: 10.1162/netn_a_00409 (PMC11675023; doi:10.1162/netn_a_00409)
Supplement: Supplementary file 1 [file netn-8-4-1439-s001.pdf]

Supplementary Materials for "CoCoNest: A Continuous Structural Connectivity-based Nested Parcellation of the Human Cerebral Cortex"

1 Additional ABCD Validation

1.1 Internal Evaluation on ABCD Data

Figure S1 shows the internal evaluation results for assessing parcellation performance using data from the ABCD study. Echoing the findings in the HCP analysis, we found that members of the CoCoNest family are simultaneously competitive with the other parcellations considered, and often shows improved performance in these internal metrics.

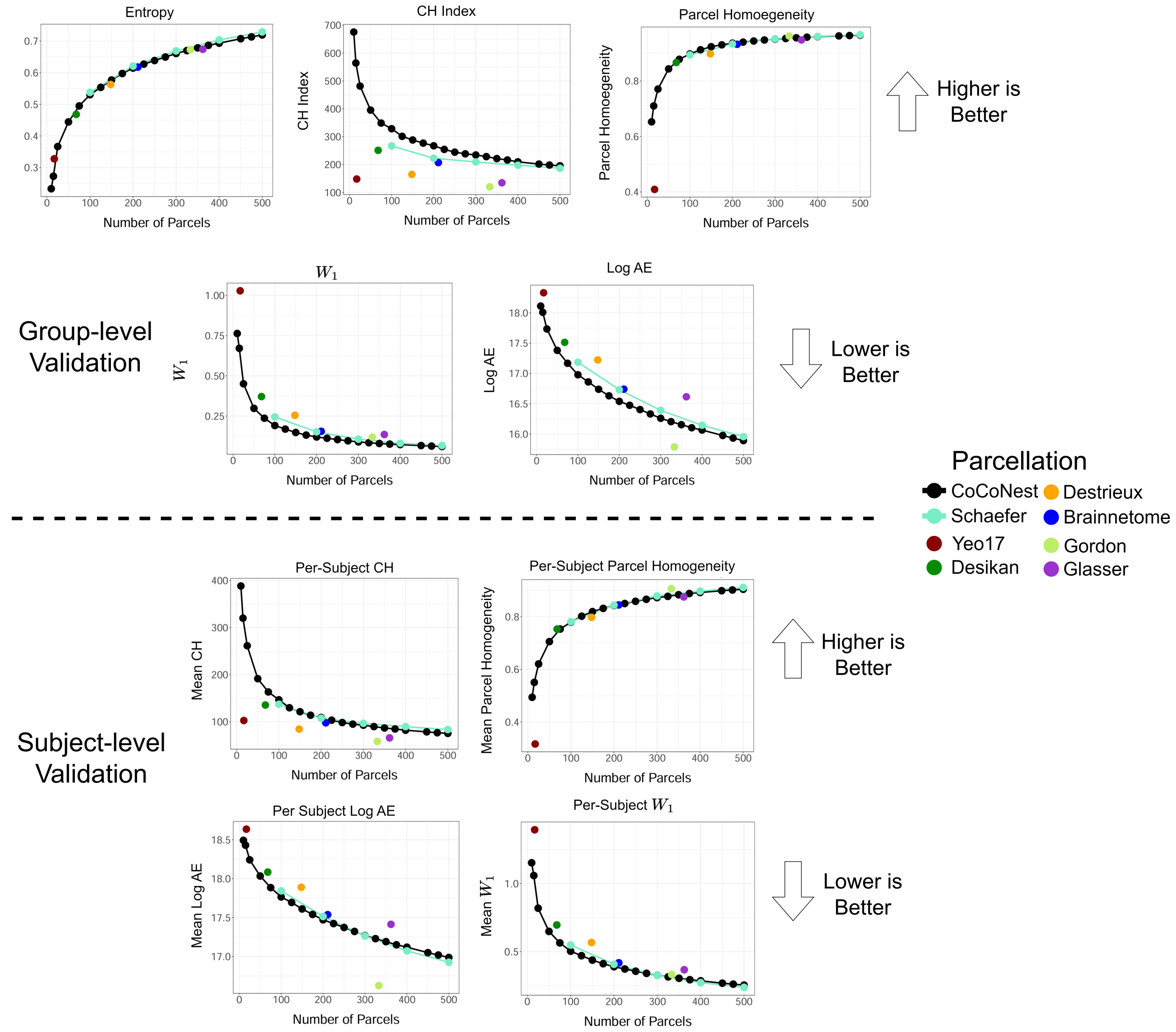

Figure S1: Results of the internal evaluation metrics for assessing parcellation performance using data from the ABCD study. The top panel shows the internal validation performed on the average of the subject-specific structural connectivity matrices of the ABCD subjects, $\bar{S}'$, while the bottom panel shows the validation performed on the subject-specific connectivity data $S'^{(i)}$. In the figure, 'CH' denotes the Calinski-Harabasz index, 'AE' denotes the Approximation Error, and 'W_1' denotes the 1-Wasserstein distance.

1.2 Validation of CoCoNest-ABCD

Parcellations are frequently applied to analyze populations that differ from those used to create the parcellations. In order to investigate the affect of this choice, we compared the performance of the CoCoNest family with a similarly derived family using the ABCD data. This was carried out using 100 ABCD subjects, who were not included in the 493 subjects used for validation. We then derived a CoCoNest family from the ABCD data, denoted CoCoNest_ABCD, following

the pipeline introduced in the paper (see Figure 1). Figures S2 and S3 show the results of the internal and external validation metrics on 493 ABCD subjects used for validation. We found that CoCoNest_ABCD shows a moderate increase in performance across the majority of the validation tasks, however, the HCP-derived CoCoNest can be used with little loss in performance.

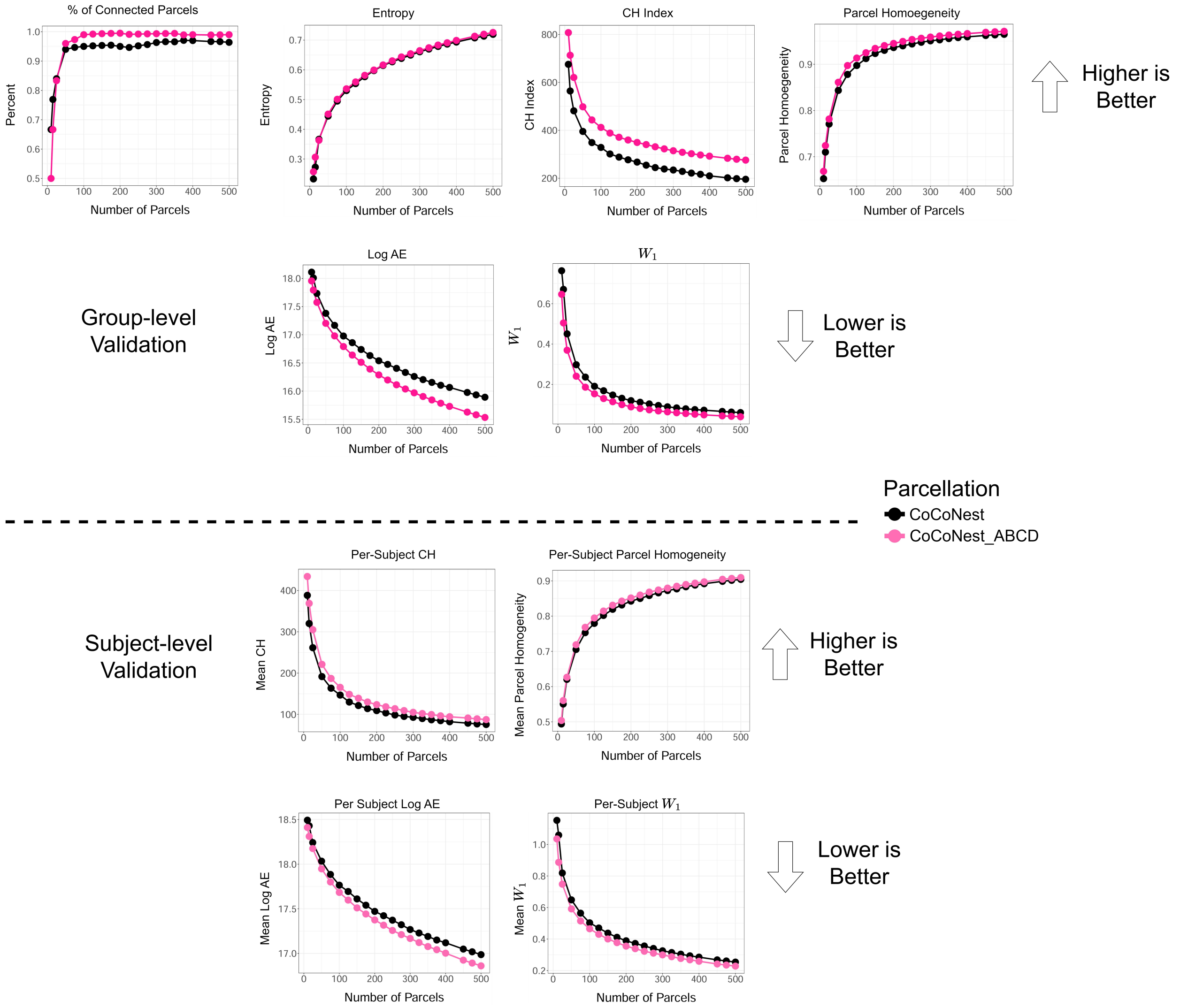

Figure S2: Results of the internal evaluation metrics for comparing the performance of CoCoNest with CoCoNest_ABCD. The top panel shows the internal validation performed on the average of the subject-specific structural connectivity matrices of the ABCD subjects, $\bar{S}'$, while the bottom panel shows the validation performed on the subject-specific connectivity data $S'^{(i)}$. In the figure, 'CH' denotes the Calinski-Harabasz index, 'AE' denotes the Approximation Error, and 'W_1' denotes the 1-Wasserstein distance.

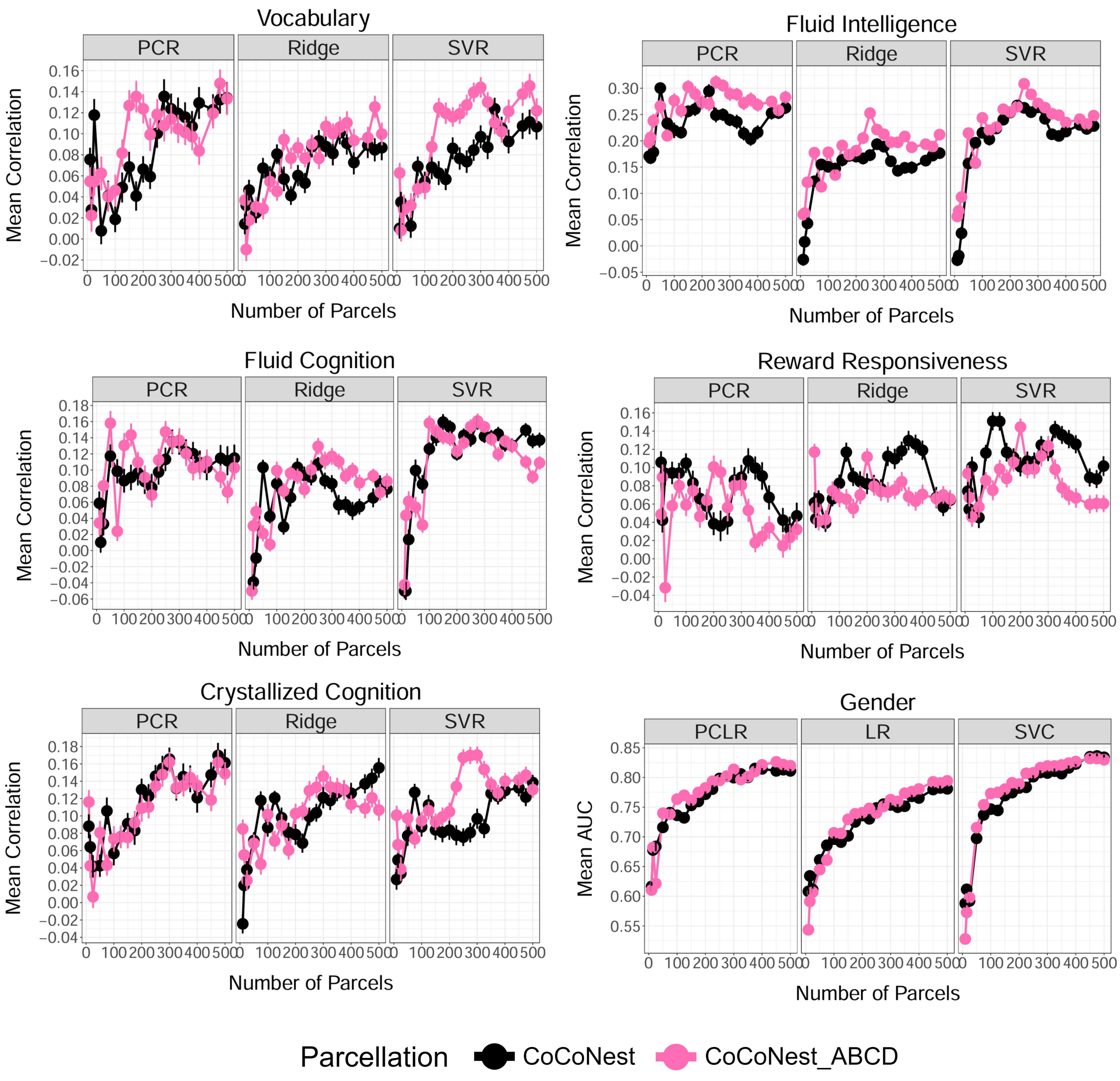

Figure S3: Results of predicting different human traits using parcel-based SC matrices generated from ABCD subject-specific SC matrices, $S^{(i)}(x, y)$, by CoCoNest and CoCoNest_ABCD. For predicting human traits, the mean predictive performance (correlation between predicted and reported values) over 50 training/testing splits is plotted vs the number of parcels in each parcellation. The error bars represent the standard error of the mean.

2 Decisions in Tree Creation and Pruning

2.1 Comparison to Horizontal Pruning

Figure S4 illustrates horizontally pruning a dendrogram to derive 8 clusters. This method of pruning is popular among hierarchical clustering implementations. However, previous work has shown that given measures of error and complexity an error-complexity pruning procedure outperforms horizontal pruning (Breiman et al., 1984; Ge and Tibshirani, 2023). To support our choice of error-complexity pruning over horizontal pruning, we used the internal and external evaluation metrics (see Figures S5 and S6) introduced in the main text. We found that our implementation of error-complexity pruning consistently outperformed horizontal pruning in these metrics across most resolutions. Additionally, Figure S7 shows visualizations of pruned subtrees derived by pruning the full CoCoNest tree with error-complexity and horizontal pruning.

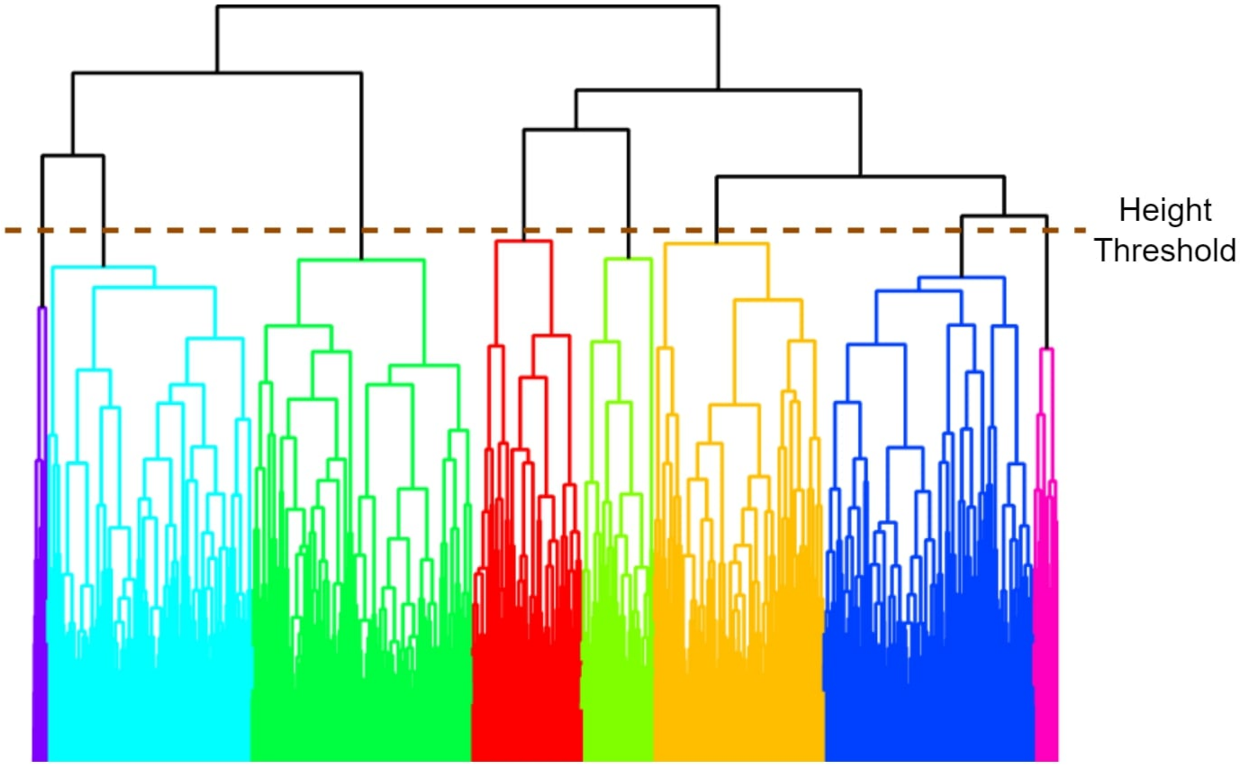

Figure S4: An example of horizontal pruning. Recall that the height of a node in the dendrogram indicates the similarity between the clusters, or parcels, being merged. The height threshold sets a limit on the maximum $D(E_i, E_j)$ allowable for merging parcels, where high values of $D(E_i, E_j)$ indicate dissimilar parcels.

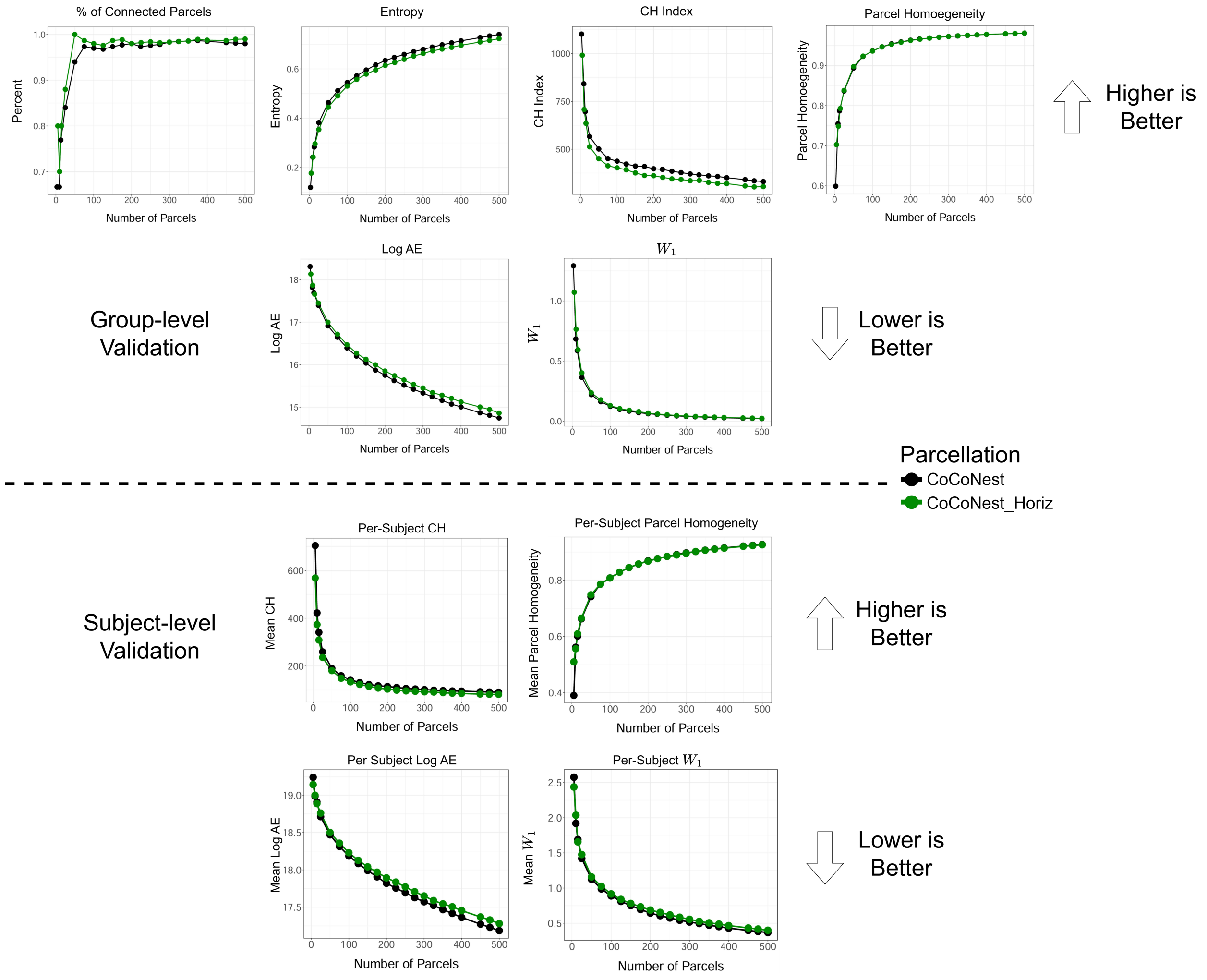

Figure S5: Results of the internal evaluation metrics for assessing the performance of error-complexity pruning (CoCoNest) versus horizontal pruning (CoCoNest_Horiz). The top panel shows the internal validation performed on the average of the subject-specific structural connectivity matrices, $\bar{S}'$, while the bottom panel shows the validation performed on the subject-specific connectivity data $S'^{(i)}$. In most of these metrics, the performance is similar with error-complexity pruning showing slightly better performance.

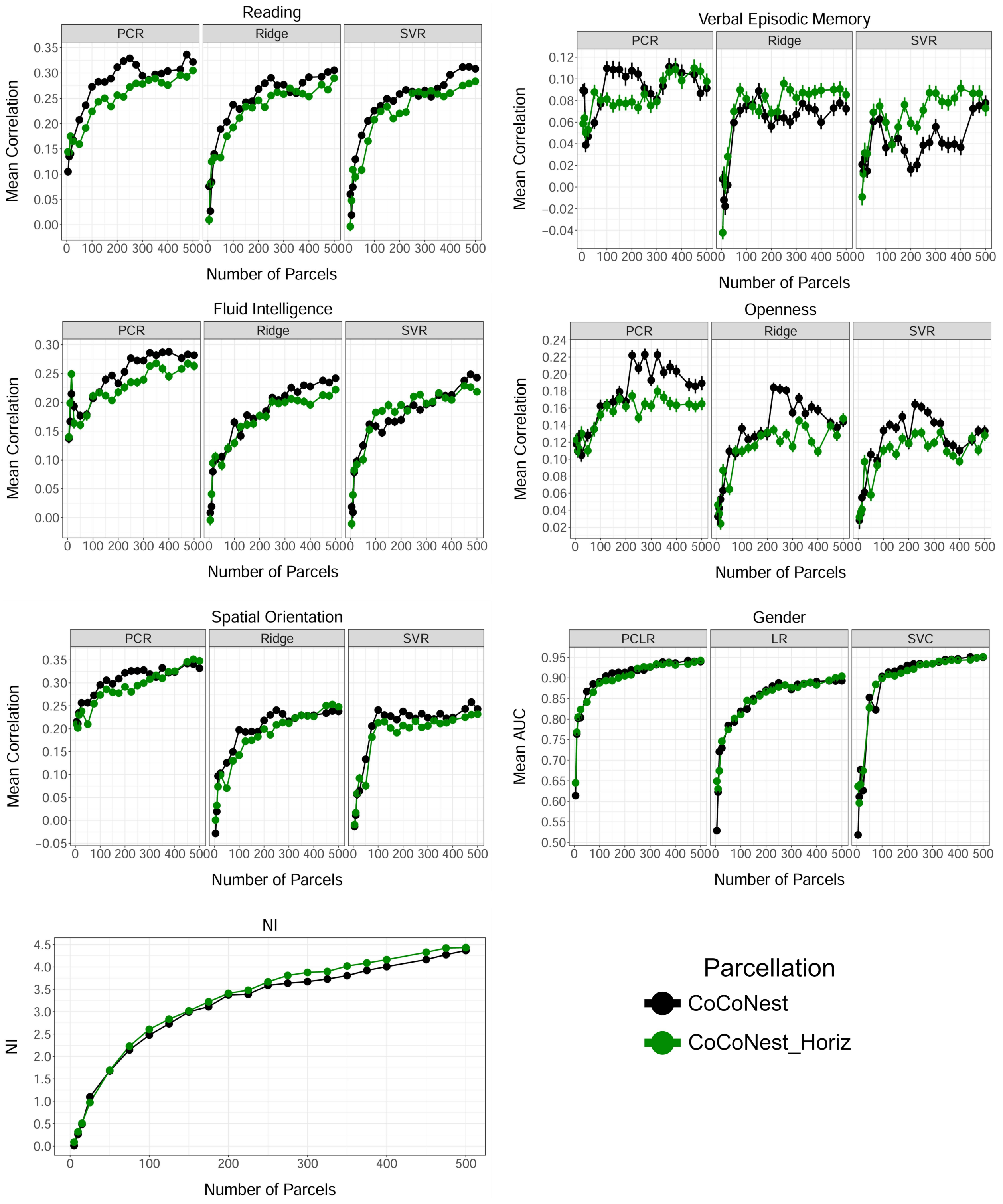

Figure S6: Results of the external evaluation metrics for assessing the performance of error-complexity pruning versus horizontal pruning. For predicting human traits, the mean predictive performance (correlation between predicted and reported values) over 50 training/testing splits is plotted vs the number of parcels in each parcellation. The error bars represent the standard error of the mean. Error-complexity pruning showed superior performance across all traits and most resolutions.

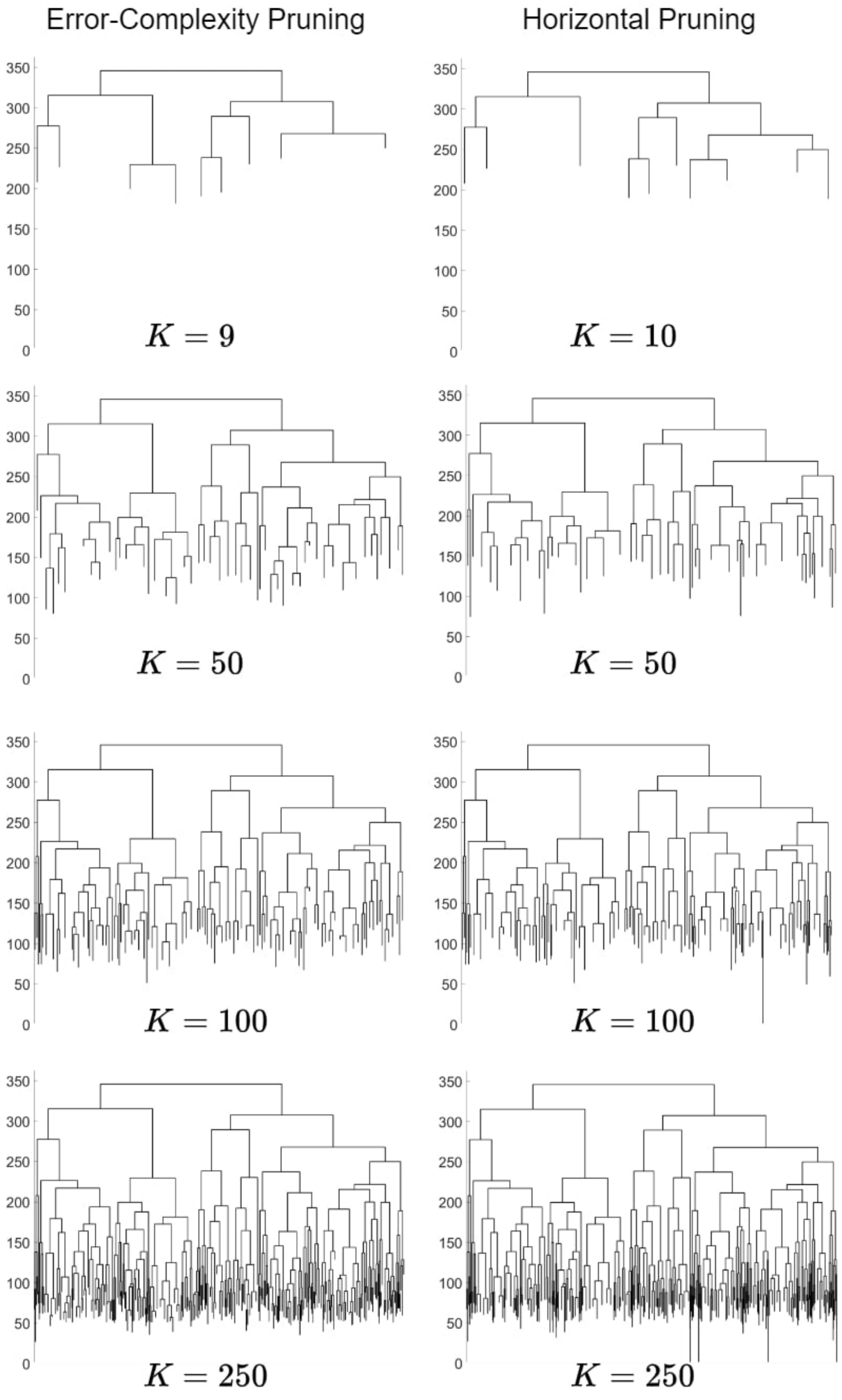

Figure S7: Visualization of subtrees of the full CoCoNest tree derived via error-complexity pruning (left) and horizontal pruning (right).

2.2 Using Different Similarity and Linkage Functions

We used Euclidean distance and average linkage to construct the full CoCoNest tree. This choice was informed by comparisons with other popular distance and linkage functions. Figures S8 and S9 shows the internal and external validation results using popular distance functions including, Euclidean distance (CoCoNest), cosine distance (CoCoNest_Cosine), and L_1 distance (CoCoNest_L_1). All distance metrics displayed similar performance in the internal evaluation metrics. We found that average linkage achieved higher performance than the other distance functions considered across most traits.

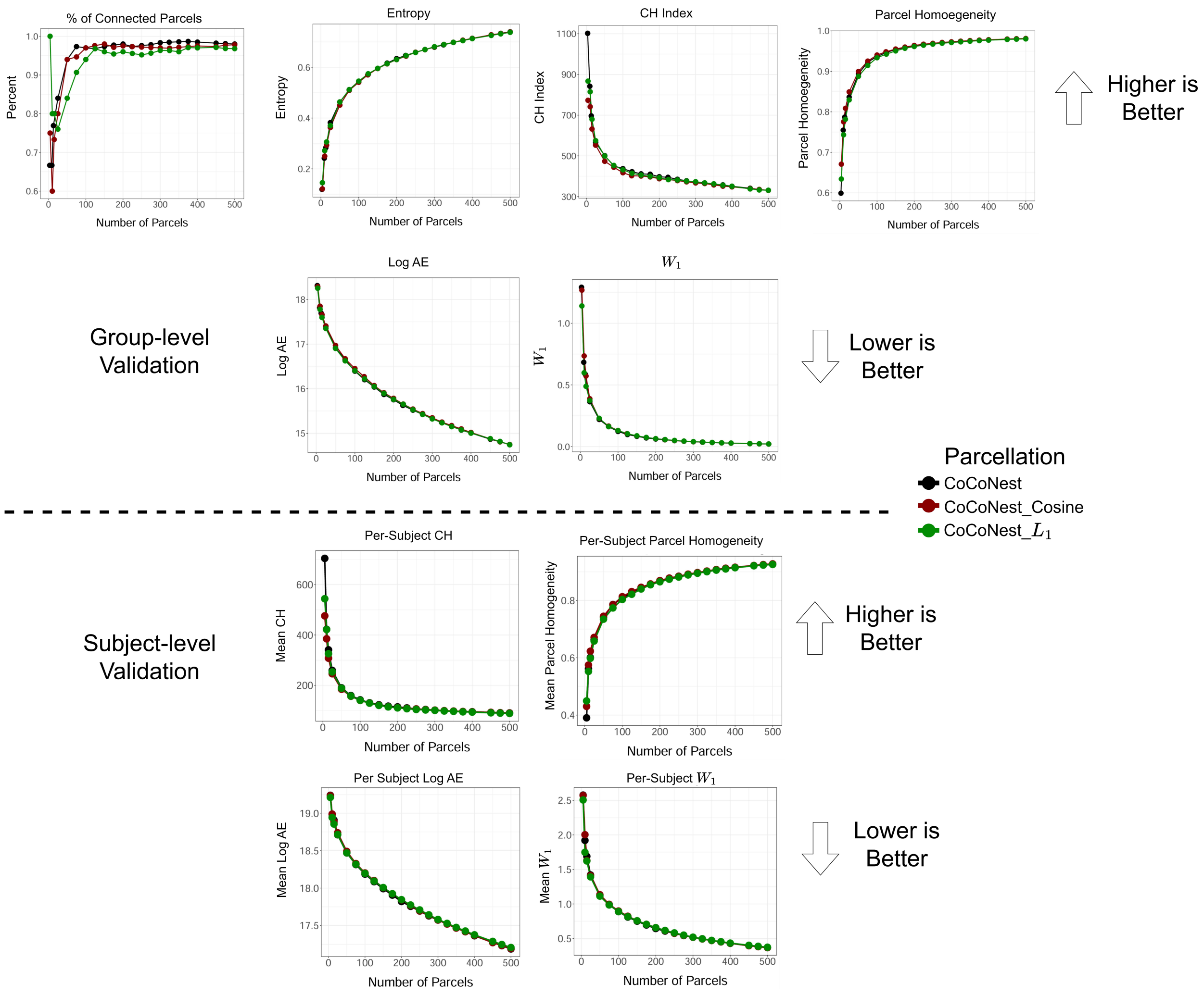

Figure S8: Results of the external evaluation metrics for assessing the performance of error-complexity pruning versus horizontal pruning. Error-complexity pruning showed superior performance across all traits and most resolutions.

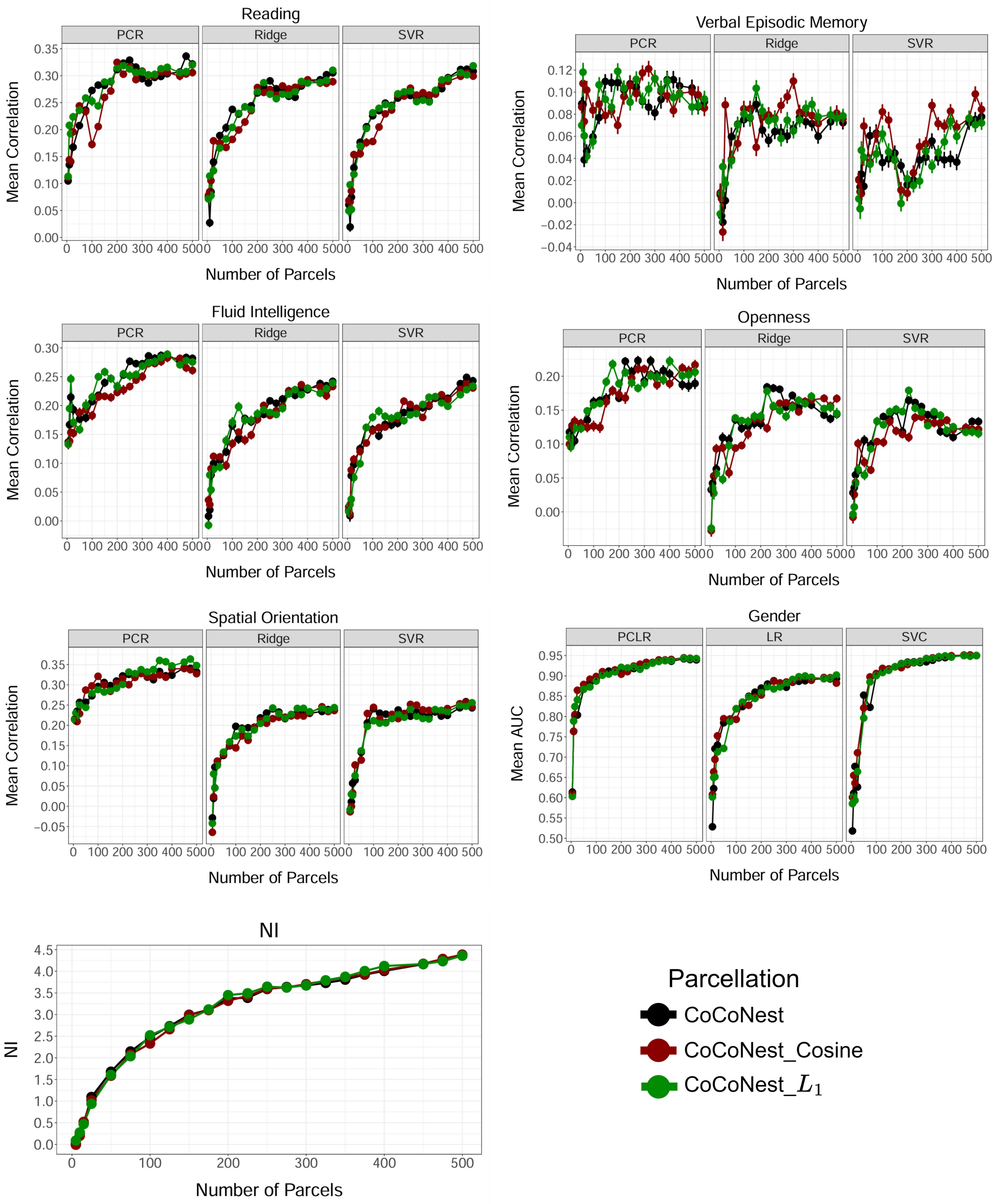

Figure S9: Results of the external evaluation metrics for assessing the performance of error-complexity pruning versus horizontal pruning. Error-complexity pruning showed superior performance across all traits and most resolutions.

Figures S10 and S11 shows the internal and external evaluation metrics for popular choices in linkage functions including, average linkage (CoCoNest), Ward's linkage (CoCoNest_Ward), complete linkage (CoCoNest_Complete), and centroid linkage (CoCoNest_Centroid). We found that the linkage functions showed similar performance across the internal evaluation metrics. Similarly, the different linkage functions showed similar performance in predicting human traits, however, we found that average linkage tends to achieve the highest performance across all of the traits.

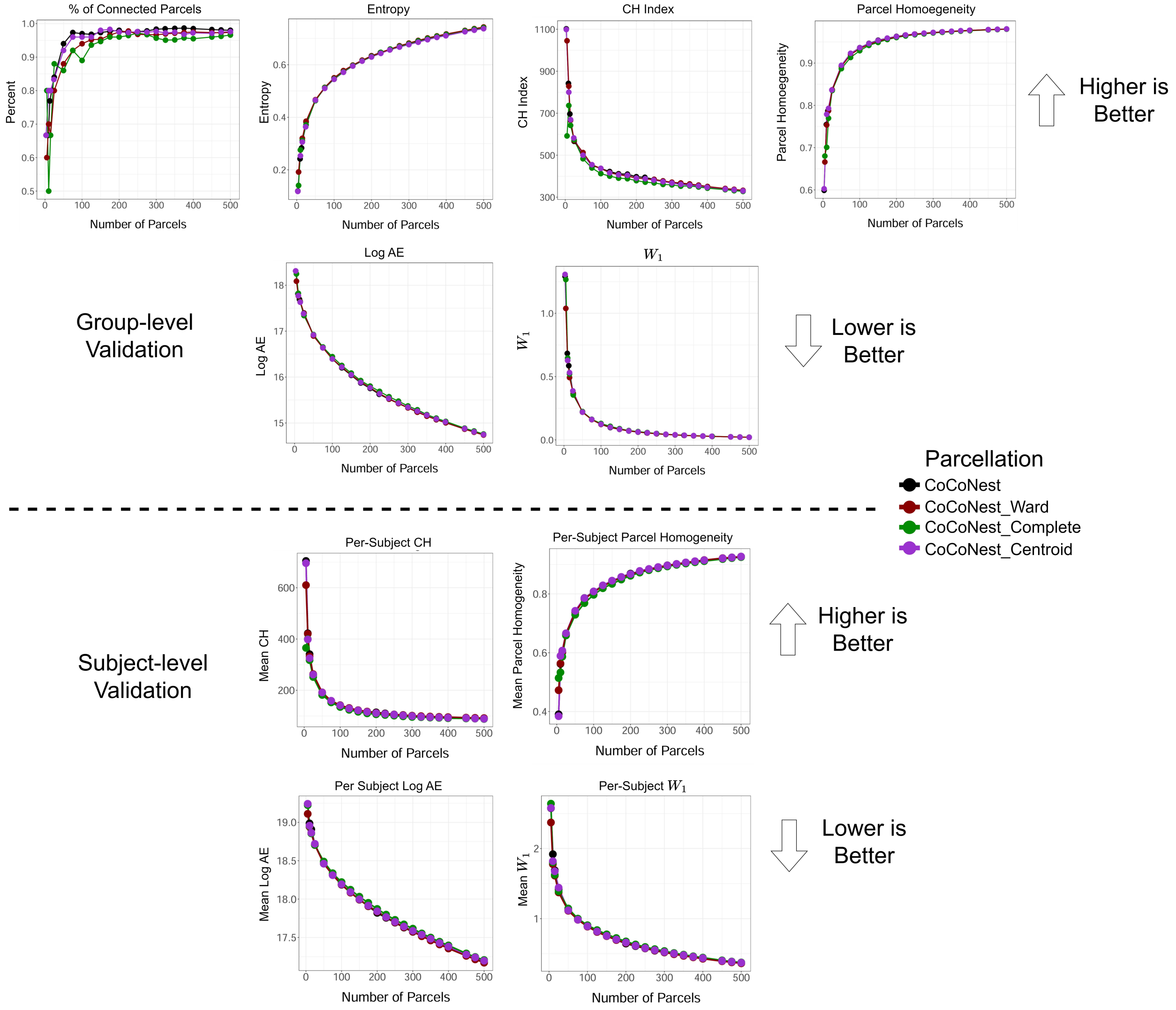

Figure S10: Results of the external evaluation metrics for assessing the performance of error-complexity pruning versus horizontal pruning. Error-complexity pruning showed superior performance across all traits and most resolutions.

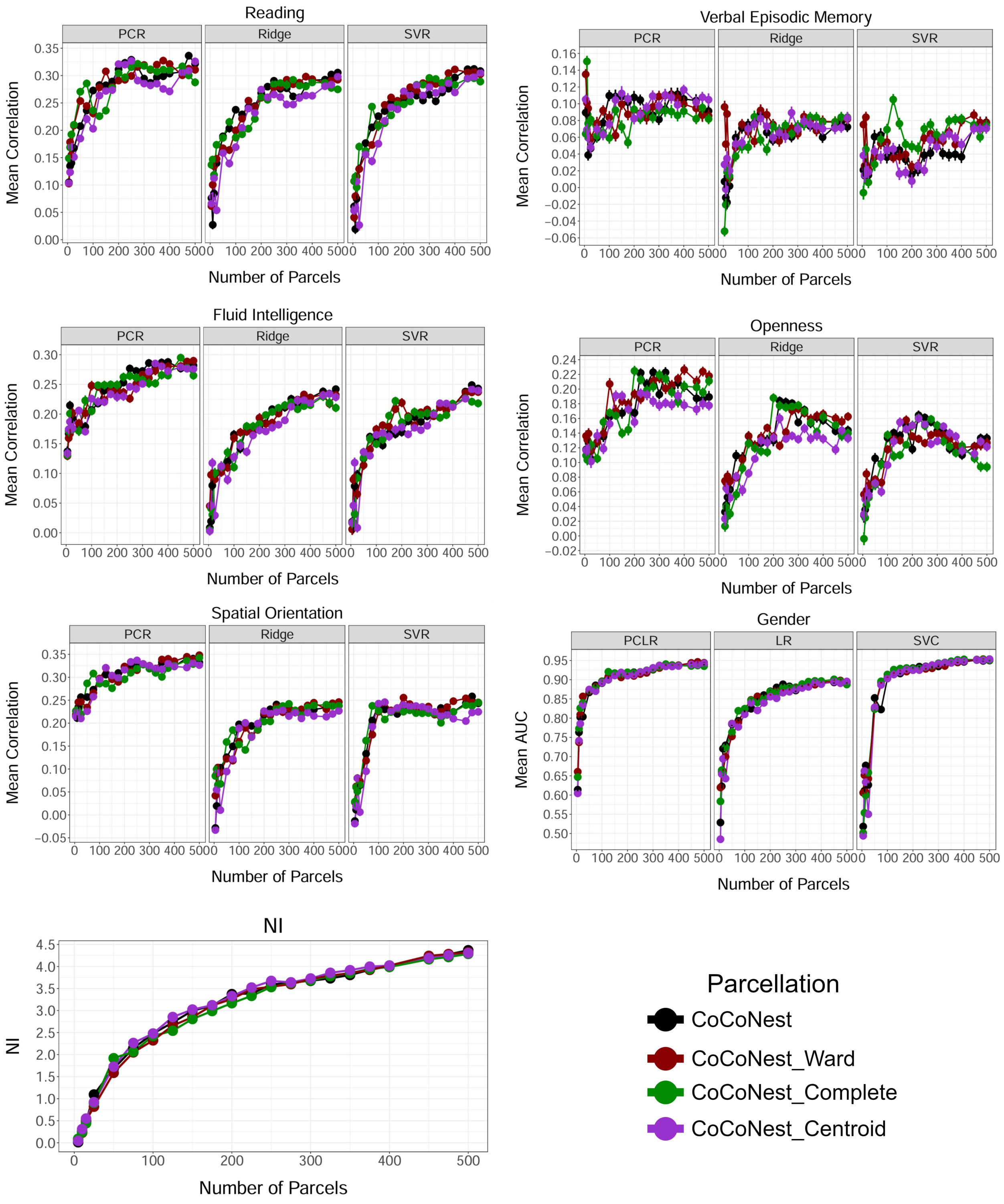

Figure S11: Results of the external evaluation metrics for assessing the performance of error-complexity pruning versus horizontal pruning. Error-complexity pruning showed superior performance across all traits and most resolutions.

3 Details on Rich Club Analysis

The presence of the rich club effect is commonly revealed through the weighted rich club coefficient. Treating $S_{\mathcal{A}}$ as a weighted network, let W^{ranked} be a vector containing all connections in $S_{\mathcal{A}}$ ranked in ascending order, $E_{>k}$ be the number of edges between nodes with degree greater than k, and $W_{>k}$ be the sum of the weighted connections between these nodes. The weighted rich club coefficient is then defined as the ratio between $W_{>k}$ and the sum of the weights associated with the $E_{>k}$ strongest connections in the entire network (Opsahl et al., 2008)

$$\phi^w(k) = \frac{W_{>k}}{\sum_{l=1}^{E_{>k}} w_l^{\text{ranked}}}. \tag{S1}$$

In other words, Equation S1 is the ratio between the sum of the weighted connections among nodes with degree greater than k and the total weighted connection they would share if they were connected by the network's strongest edges (Opsahl et al., 2008). Since nodes of higher degree are likely to be interconnected due to chance alone, $\phi^w(k)$ is commonly normalized relative to a collection of similar random networks (Heuvel and Sporns, 2011; Colizza et al., 2006). To do this, for each $S_{\mathcal{A}}$ considered, we created 1000 random networks, with the same sequence and degree distribution (Rubinov and Sporns, 2010). For each of these random networks, the rich club coefficients were computed. We denote these coefficients as $\phi_{\text{random}}(k)$. The normalized rich club coefficient (Opsahl et al., 2008) is then defined as

$$\phi_{\text{norm}}(k) = \frac{\phi(k)}{\phi_{\text{random}}(k)}. \tag{S2}$$

A normalized rich club coefficient greater than one for successive values of k indicates the presence of the rich club effect.

Figure 11 shows the results of the multi-scale network analysis using three members of the CoCoNest family. Additionally, we carried out a network-based analysis of the structural connectome using three of the considered parcellations including, the Brainnetome, Glasser, and Schaefer parcellations. Figure S12(a) shows the weighted degree of each parcel on the cortical surface. Similar to the multi-scale network analysis with CoCoNest, parcels in the frontal and parietal lobes showed lower weighted degrees than parcels in the occipital lobe. Figure S12(c) shows the rich-club nodes on the cortical surface. Each parcellation considered found rich club parcels in similar locations as the CoCoNest family members in the frontal and occipital lobes. However, we found that the Brainnetome parcellation and the Glasser parcellation found rich club parcels in the more anterior portion of the frontal lobe than the CoCoNest members and the Shaefer parcellation.

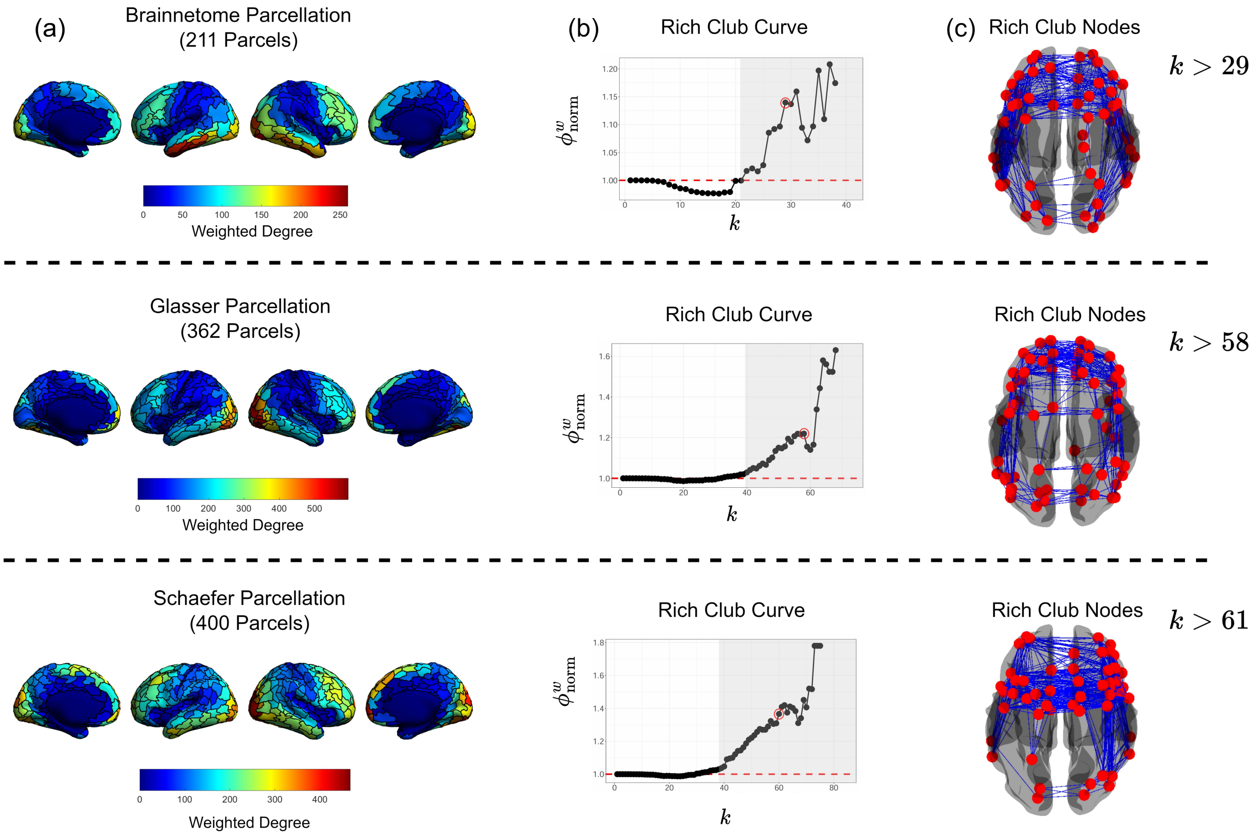

Figure S12: Network analysis with several popular parcellations including the Brainnetome, Glasser, and Schaefer parcellations. Panel (a) displays the weighted degree of each parcel on the cortical surface. Panel (b) shows the rich club curve. The region shaded in gray indicates the rich club regime where ϕ^w_{norm} remains greater than one for successive values of k. Panel (c) displays the rich club nodes on the cortical surface (represented as red balls), along with the connections between them (represented as blue line segments).

4 Examples of Non-connected Clusters

Downsampling from the high-resolution white matter surface meshes (See Section 4) introduced small errors in the parcel assignments. As seen in Figure 4, this led to several parcels, in known contiguous parcellations, being considered non-connected. Examples are shown in Figure S13. 16 parcels in the Yeo-17 parcellation, a single parcel in the Desikan parcellation, 18 parcels in the Destrieux parcellation, 12 parcels in the Brainnetome atlas, and 42 parcels in the Glasser parcellation were identified as non-connected by the depth-first search algorithm.

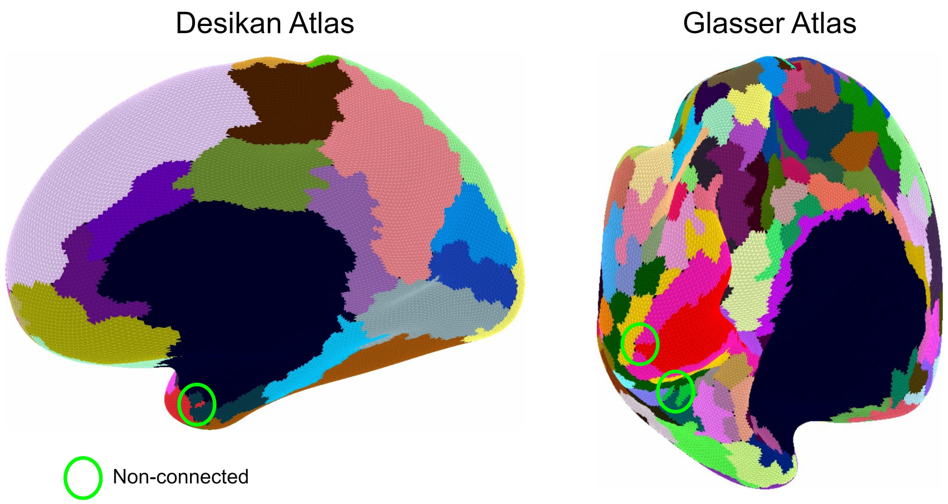

Figure S13: Examples of parcels considered non-contiguous by the depth-first search algorithm visualized on the inflated white surface.
